# Acupuncture for emotional symptoms in patients with functional gastrointestinal disorders: A systematic review and meta-analysis

**DOI:** 10.1371/journal.pone.0263166

**Published:** 2022-01-27

**Authors:** Ling Wang, Jin Xian, Mi Sun, Xue Wang, Xiaoming Zang, Xin Zhang, Huijuan Yu, Qi-Wen Tan

**Affiliations:** 1 Shandong University of Traditional Chinese Medicine, Jinan, Shandong, China; 2 Affiliated Hospital of Shandong University of Traditional Chinese Medicine, Jinan, Shandong, China; Lluita contra la SIDA Foundation - Germans Trias i Pujol University Hospital, SPAIN

## Abstract

**Background:**

Patients with functional gastrointestinal disorders (FGIDs) also often have emotional symptoms, such as anxiety and depression. The main drugs used for the treatment of FGIDs mainly target single gastrointestinal symptoms and are not effective in regulating emotional symptoms. Evidence has shown that acupuncture can relieve gastrointestinal symptoms in FGIDs patients, but there is no high-quality evidence to show that acupuncture can relieve psychological symptoms in these patients.

**Objectives:**

To systematically evaluate the clinical efficacy and safety of acupuncture for emotional symptoms in patients with FGIDs.

**Methods:**

Randomized controlled trials (RCTs) published from database inception through July 31, 2021, were retrieved from three English-language databases (PubMed, the Cochrane Central Register of Controlled Trials, and Embase) and five Chinese-language databases (the China National Knowledge Infrastructure, Wanfang, VIP, Chinese Biomedical, and TCM Literature Analysis and Retrieval databases). RCTs that compared acupuncture with sham acupuncture and pharmacotherapy were included in this study. The score on the depression or anxiety scale after treatment were considered as primary outcomes. The ‘meta’ package (version 4.19–0) in RStudio 1.1.463 was used to analyse the data.

**Results:**

A total of 2151 patients from 24 RCTs were included in this study. Compared with sham acupuncture, acupuncture was not significantly better at relieving anxiety (standardized mean difference [SMD] -0.35, 95% CI −1.05 to 0.33) and depression (SMD -0.32, 95% CI −0.71 to 0.07) symptoms. Compared with pharmacotherapy, acupuncture was significantly better at relieving anxiety (SMD -0.64, 95% CI -0.93 to -0.35) and depression (SMD -0.46, 95% CI -0.69 to -0.22) symptoms.

**Conclusions:**

This meta-analysis found that acupuncture can alleviate emotional symptoms in FGID patients better than pharmacotherapy. However, it is not clear whether this effect is based on the placebo effect, specific effect or nonspecific effect of acupuncture. The evidence should be proven by rigorously designed RCTs in the future.

**PROSPERO registration number:**

CRD42021271899.

## 1. Introduction

Functional gastrointestinal diseases (FGIDs), which are defined as gut-brain interaction disorders [1], are the most common types of gastrointestinal diseases [2]. In a large multinational study, researchers found that more than 40% of people worldwide have FGIDs [3]. FGIDs are characterized by morphological and physiological abnormalities, including dysmotility, visceral hypersensitivity, changes in mucosal and immune function, changes in the gut microbiota and processing-related changes in the central nervous system. Irritable bowel syndrome (IBS), functional dyspepsia (FD), functional constipation (FC) and functional diarrhoea (FDr) are the most common FGIDs and may affect quality of life and health care resource utilization. Patients with FGIDs also often have emotional symptoms, such as anxiety and depression. These symptoms often influence each other, leading to recurrence or aggravation of the disease. Therefore, it is important to pay attention to emotional symptoms in patients with FGIDs. The main drugs used for the treatment of FGIDs are kinetic agents, proton pump inhibitors, anticholinergics and antidiarrhoeal drugs. However, these drugs mainly target single gastrointestinal symptoms and are not effective at regulating emotional symptoms. Some studies have shown that antidepressants can be administered to FGID patients [4, 5], but their use is limited because of their adverse reactions.

Complementary and alternative medicine (CAM) is a unique group of medical practices and products, and a large number of patients who suffer from FGIDs turn to CAM to control their symptoms; many of these patients are happy with the therapeutic results [6]. Acupuncture is an important part of CAM and has been practised for thousands of years in China. Evidence has shown that acupuncture can relieve gastrointestinal symptoms of FGIDs [7] and emotional symptoms such as depression [8] and anxiety [9]. However, most studies that have analysed acupuncture for FGIDs focused on gastrointestinal symptoms rather than psychological symptoms. Several studies have shown that acupuncture may relieve gastrointestinal and psychological symptoms better than controls [10–12], but other studies have shown discordant results [13, 14]. Hence, in this study, we summarized evidence from randomized controlled trials (RCTs) and evaluated the effectiveness of acupuncture on relieving emotional symptoms in patients with FGIDs.

## 2. Methods

### 2.1 Criteria for considering studies for this review

We included RCTs with parallel groups and excluded conference abstracts, editorials, reviews and case reports or case series, as well as publications reporting duplicate data. All participants included in the study were adults diagnosed with FGIDs, such as FD, IBS, FC or FDr, according to the Rome criteria [1, 15]. The experimental group included patients treated with acupuncture, defined as needle insertion at an acupuncture point, including body acupuncture (manual/electro), ear acupuncture and scalp acupuncture. Studies including other types of acupoint stimulation without needle insertion, such as laser stimulation and transcutaneous electroacupuncture therapy, were excluded. The control group included patients who had been treated with sham acupuncture or medication. The included studies reported the results of validated screening scales for anxiety or depression, such as the self-rating anxiety scale (SAS), self-rating depression scale (SDS), Hamilton rating scale for anxiety (HAM-A), Hamilton rating scale for depression (HAM-D), patient health questionnaire-9 scale (PHQ-9) or generalized anxiety disorder-7 scale (GAD-7). Studies that reported total scores of depression and anxiety obtained through tools such as the hospital anxiety depression scale were excluded.

### 2.2 Search strategy and study selection

Studies included in the review were retrieved from three English-language (PubMed, the Cochrane Central Register of Controlled Trials, and Embase) and five Chinese-language databases (the China National Knowledge Infrastructure, Wanfang, VIP, Chinese Biomedical and TCM Literature Analysis and Retrieval databases); RCTs published from the time of database inception to July 31, 2021, were retrieved. The search procedure is shown in S1 Table in S1 File. In addition, we searched Google Scholar and the ChiCTR clinical trial registration platform and manually searched journal articles and conference proceedings in the library of Shandong University of Traditional Chinese Medicine.

Two investigators (JX and LW) independently screened the study titles and abstracts and full texts when necessary. A third reviewer (XZ) made the final decision when a disagreement occurred between the two initial investigators.

### 2.3 Data extraction and quality assessment

Two investigators (MS and XW) independently extracted the data using a predesigned form. The name of the author, year of publication, inclusion and exclusion criteria, number of patients, type of acupuncture, acupoints, treatment used in the control group and outcome measures were recorded. We used the GetData Graph Digitizer to extract numerical data from figures. Data that could not be extracted from the original publications were requested from the corresponding authors or searched for in other reviews. Two investigators (JX and LW) independently assessed the risk of bias using a revised tool to assess the risk of bias in randomized trials (RoB 2) [16], and the RCTs were classified as having ‘low risk’, ‘some concerns’ or ‘high risk’. A third investigator (HY) resolved any disagreements.

### 2.4 Statistical analysis

We calculated risk ratio (RR) with 95% confidence intervals (CI) for dichotomous data and standardized mean difference (SMD) with 95% CI for continuous data. The primary outcome measure was the score on the depression or anxiety scale after treatment. The heterogeneity of the studies was evaluated using the χ2 test and I^2^ statistic. Fixed-effect and random-effect models were used for the meta-analyses. We used the estimates of the random-effects model when high heterogeneity (I^2^≥50% or p<0.1) was present; otherwise, we used the fixed-effect model estimates. We preferred analysed the data based on the intention-to-treat sample. In addition, patients treated with acupuncture in some RCTs were divided into different groups according to acupuncture point. Since the acupoints used in each group were commonly used acupuncture points for the treatment of FGIDs, we combined the results of different acupuncture groups to obtain information representative of the real clinical efficacy. To explore possible clinical heterogeneity, we performed subgroup analyses by disease, type of acupuncture and whether acupoints for tranquilization were included. Sensitivity analysis was performed with the leave-1-out function to confirm the robustness of our results. Publication bias was assessed using contour-enhanced funnel plots and Egger’s test for outcomes when at least 10 trials were included. Quality of evidence was summarized with the Grading of Recommendations Assessment, Development, and Evaluation (GRADE) approach and is presented in ‘Summary of findings’ tables [17]. All the analyses in this review were conducted using the ‘meta’ package (version 4.19–0) [18] in R Studio 1.1.463.

## 3. Results

### 3.1 Study selection

**Fig 1:**
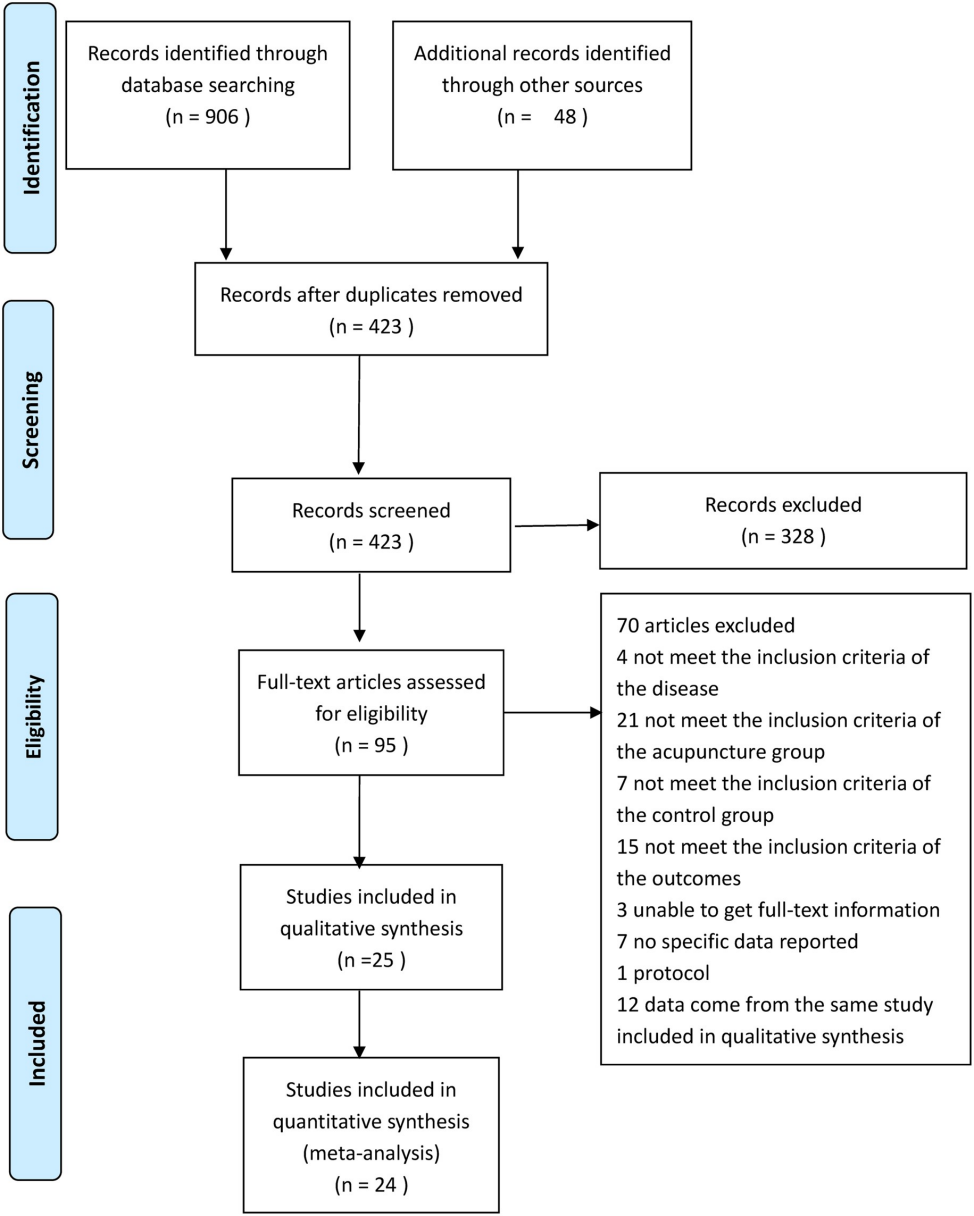
PRISMA flow diagram.

A total of 954 reports were retrieved through database searching; 531 duplicate publications and 328 were excluded after title and abstract screening. After reviewing the full texts of the remaining studies, 25 reports from 24 studies that met the inclusion criteria were included in the systematic review or qualitative analysis (Fig 1).

### 3.2 Characteristics of the included RCTs

We analysed a total of 2151 patients from 24 RCTs [10–14, 19–38]. Two of the included RCTs were multicentre studies, and 22 were single-centre studies. The sample sizes of the studies ranged from 34 to 348, and the duration of treatment lasted 2–10 weeks. Regarding treatment groups, 17 studies used electroacupuncture, and 7 studies used manual acupuncture. Regarding control groups, 20 studies used pharmacotherapy, and 4 studies used sham acupuncture. There were two four-arm trials [22, 26] and two three-arm trials [13, 14, 37]. Seventeen studies reported SAS scores, 17 studies reported SDS scores, 4 studies mentioned HAM-A scores, 5 studies reported HAM-D scores, and 1 study reported PHQ-9 and GAD-7 scores. Detailed characteristics of the research are summarized in Table 1.

**Table 1:** Characteristics of the included studies.

Studys	Disease	Sample Size		Mean Age±SD		Sex (Male/Female)		Experimental Group		Control Group	Treatment duration	Outcome
		E	C	E	C	E	C	Acupoints	Method of acupuncture			
Yang2011	Functional dyspepsia	30	30	37.6± 9.8	38.4 ±10.1	13/17	15/15	Zusanli, Neiguan	EA	Domperidone	2W	SAS, SDS
Li2011	Irritable bowel syndrome	35	35	39.1±11.80	37.93±11.45	15/20	18/17	Tianshu, Zusanli, Shangjuxu, Sanyinjiao, Taichong, Baihui, Yintang	EA	Pinaverium bromide	4W	SAS, SDS
Chen2012	Irritable bowel syndrome	30	34	40.5±8.75	41.9±10.01	11/19	14/20	Baihui, Shenting, Neiguan, Shenmen, Zhongwan, Tianshu, Qihai, Sanyinjiao, Taichong	EA	Bacillus licheniformis + Flupentixol and Melitracen Tablets	4W	HAMA, HAMD
Peng2013	Irritable bowel syndrome	20	20	47.5±3.2	45.8±4.0	8/12	10/10	Zusanli, Tianshu	EA	Macrogol 4000 Powders + Combined Bacillus Subtilis and Enterococcus Faecium Granules with Multivitamines Live	4W	SAS, SDS
Zhong2013	Functional constipation	170	62	43.85±17.79	41.97±18.07	33/139	13/49	1: Tianshu, Dachangshu	EA	Mosapride	4W	SAS, SDS
								2: Quchi, Shangjuxu				
								3: Tianshu, Dachangshu, Quchi, Shangjuxu				
Wu2013	Functional constipation	23	18	27.91±8.91	28.39±9.68	8/15	6/12	Quchi, Shangjuxu	EA	Mosapride	4W	SAS, SDS
Xiong2014	Functional constipation	67	41	NR	NR	NR	NR	Quchi, Shangjuxu	EA	Mosapride	4W	SAS, SDS
Zheng2014	Irritable bowel syndrome	261	87	41.25±16.99	42.29±18.3	138/118	52/34	1: Tianshu, Dachangshu	EA	Loperamide Hydrochloride	4W	SAS, SDS
								2: Quchi, Shangjuxu				
								3: Tianshu, Dachangshu, Quchi, Shangjuxu				
Tian2014	Functional diarrhoea	37	16	39.77±15.44	39.27±16.88	NR	NR	Tianshu, Dachangshu	EA	Loperamide Hydrochloride	4W	SAS, SDS
Da2014	Functional constipation	18	16	51.22±22.84	38.38±19.61	4/14	2/14	Tianshu, Fujie, Shangjuxu	EA	SA	8W	SAS, SDS
Jin2015	Functional dyspepsia	28	28	49.29±10.32	48.25±11.40	11/17	10/18	Zusanli, Taixi, Zulinqi, Neiguan, Shenmen	MA	SA	4W	SAS, SDS
Yuan2015	Functional dyspepsia	31	32	44.21±21.12	39.21±25.12	13/18	15/17	Gongsun, Neiguan	MA	Domperidone	4W	HAMA, HAMD
Lian2016	Functional diarrhoea	32	30	33.85±12.55	31.60±11.56	14/18	11/19	Tianshu, Dachangshu, Quchi, Shangjuxu	EA	Loperamide Hydrochloride	4W	SAS
Du2016	Functional dyspepsia	48	47	44.89±10.12	43.28±12.05	13/35	15/32	Shenque, Sishencong, Shenmen, Baihui, Zusanli, Zhongwan, Neiguan	EA	Domperidone	4W	HAMD
Ding2016	Functional diarrhoea	48	19	43.19±17.08	41.37±16.74	28/17	8/11	Tianshu, Dachangshu	1: EA with strong stimulation	Loperamide Hydrochloride	4W	SAS, SDS
									2: EA with little stimulation			
Li2017	Irritable bowel syndrome	79	39	46.3±13.2	48.9±12.4	42/34	17/16	Baihui, Yintang, Tianshu, Zusanli, Shangjuxu, Sanyinjiao, Taichong	MA	Pinaverium bromide	6W	SAS, SDS
Nie2017	Functional diarrhoea	53	53	46.4±10.8	46.1±10.6	29/24	32/21	Tianshu, Shangjuxu	MA	Loperamide Hydrochloride	4W	SAS, SDS
Shen2018	Functional dyspepsia	32	32	45.71±11.26	44.21±11.59	7/27	6/28	Baihui, Yintang, Neiguan, Zhongwan, Tianshu, Qihai, Zusanli, Sanyinjiao, Taichong	EA	Mosapride	4W	HAMA, HAMD
Zhong2018	Irritable bowel syndrome	60	30	31.12±12.74	30.22±13.99	35/25	21/9	1: Quchi, Shangjuxu	EA	Loperamide Hydrochloride	4W	SAS, SDS
								2: Quchi, Shangjuxu, Tianshu, Dachangshu				
Mak2019	Irritable bowel syndrome	40	40	50.85±11.57	50.83±14.15	20/20	18/22	Neiguan, Shenmen, Zusanli, Shangjuxu, Sanyinjiao, Taichong, Baihui, Yintang	EA	SA	10W	PHQ-9, GAD-7
Meng2019	Irritable bowel syndrome	35	35	39.3±11.5	38.4±13.5	16/19	13/22	Taichong, Zusanli, Shangjuxu, Sanyinjiao, Tianshu, Baihui, Yintang	MA	Pinaverium bromide	4W	SDS
Chen2019	Functional dyspepsia	35	35	40.97±11.70	43.59±12.4	11/22	14/20	Zhongwan, Tianshu, Zusanli, Neiguan, Baihui, Taichong	MA	SA	4W	SAS, SDS
Xu2020	Functional dyspepsia	31	33	35.3±19.1	35.4±15.3	6/25	6/27	Quchi, Shangjuxu	EA	1: Mosapride	4W	SAS, SDS
										2:SA+Mosapride		
Yang2020	Irritable bowel syndrome	20	20	55.0±5.4	54±6.1	12/8	10/10	Neiguan, Tianshu, Sanyinjiao, Zusanli, Shangjuxu, Taichong, Yintang	MA	Montmorillonite powder + Flupentixol and Melitracen Tablets	2W	HAMA, HAMD

E: Experimental Group; C: Control Group; MA: Manual acupuncture; EA: Electroacupuncture; SA: Sham acupuncture; NR: Not report; SAS: Self-Rating Anxiety Scale; SDS: Self-Rating depression Scale; HAMA: Hamilton Rating Scale for Anxiety; HAMD: Hamilton Rating Scale for Depression; PHQ-9: Patient Health Questionnaire-9 items; GAD-7: Generalized Anxiety Disorder-7 item

### 3.3 Risk of bias assessment

Based on the revised tool to assess the risk of bias in randomized trials (RoB 2) [16], the overall risk of bias in 3 studies was low, in 20 studies was of some concern and in 1 study was high. The randomization process, deviations from intended interventions and measurement of the outcome were the main causes of risk of bias. S1 and S2 Figs in S1 File summarize the quality evaluations of the included studies.

### 3.4 Acupuncture versus sham acupuncture

Four RCTs compared acupuncture with sham acupuncture, and all the sham acupuncture groups received minimal acupuncture (pierced the skin 2–3 mm) at nonacupoints.

#### 3.4.1 Anxiety

**Fig 2:**
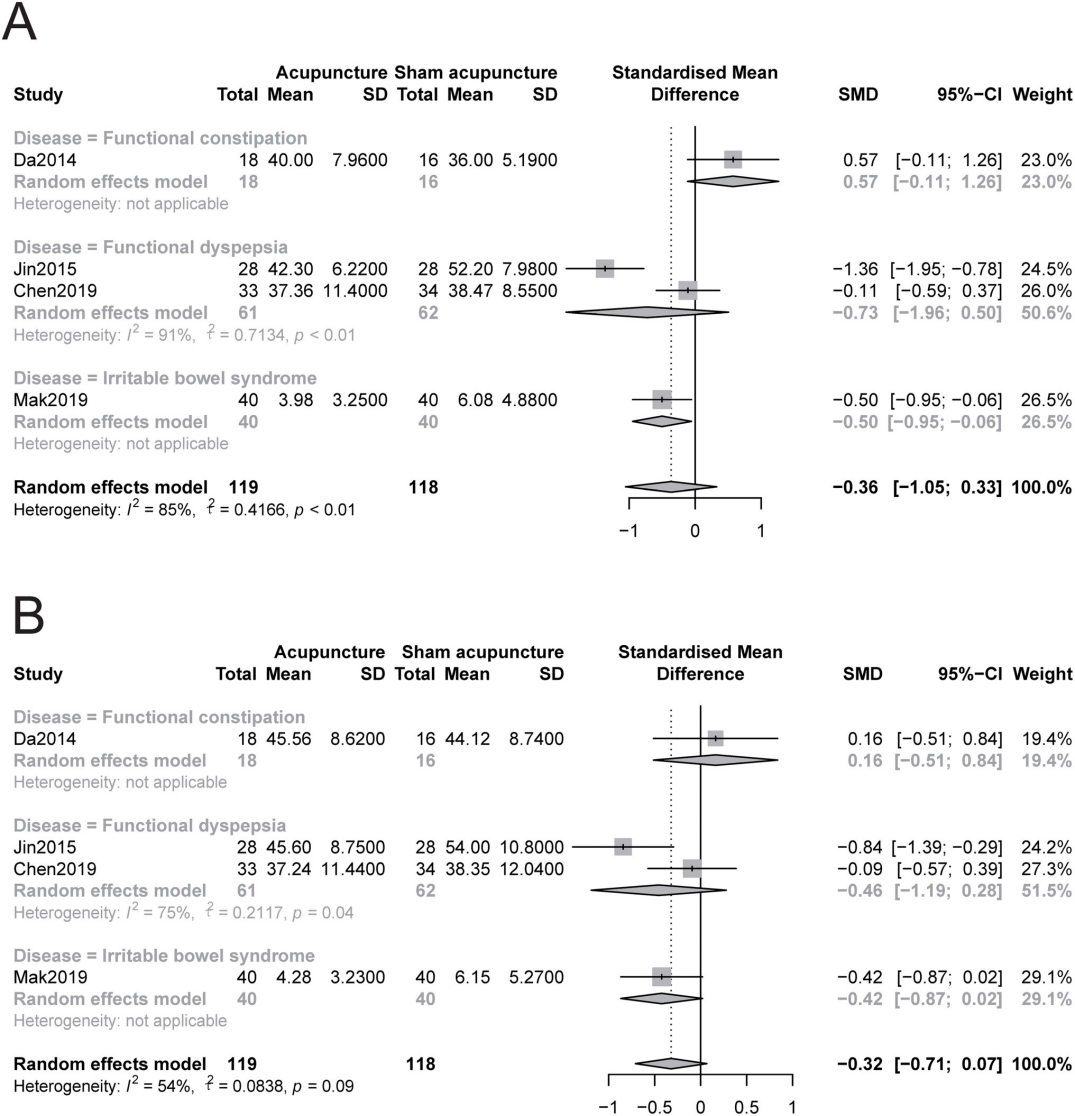
Forest plot of comparison: Acupuncture versus sham acupuncture, outcome: Severity of anxiety and depression at the end of treatment. A: anxiety, B: depression.

The pooled results indicated that acupuncture was not significantly better at relieving anxiety symptoms than sham acupuncture (SMD -0.35, 95% CI −1.05 to 0.33, I2 = 85%, Fig 2A). For better visualization, we also constructed a drapery plot [39] (S3 Fig in S1 File). The GRADE quality of the evidence was low (Table 2). Sensitivity analysis confirmed the robustness of the results (S4 Fig in S1 File). In addition, the subgroup analysis based on disease showed that acupuncture may be superior to sham acupuncture in relieving anxiety symptoms in patients with IBS (SMD -0.5, 95% CI -0.95 to -0.06), but there was no significant relief of anxiety symptoms in patients with FD, FC or FDr (Fig 2A). The subgroup analyses based on acupoints and type of acupuncture showed that the inclusion of acupoints for tranquilization or the type of acupuncture did not explain the heterogeneity. It also showed that acupuncture was not more effective than sham acupuncture in reducing anxiety symptoms (S5 and S6 Figs in S1 File).

**Table 2:** Summary of findings.

Acupuncture for emotional disorders in patients with functional gastrointestinal disorders						
Outcomes	Illustrative comparative risks* (95% CI)		Relative effect (95% CI)	No of Participants (studies)	Quality of the evidence (GRADE)	Comments
	Assumed risk	Corresponding risk				
	Control	Acupuncture				
**Acupuncture vs.sham acupuncture**		SMD 0.36 lower		237 (4 studies)	⊕⊕⊝⊝ **low**^1^^,^^2^	As a rule of thumb, 0.2 SMD represents a small difference, 0.5 moderate, and 0.8 large.
Anxiety		(1.05 lower to 0.33 higher)				
**Acupuncture vs. sham acupuncture**		SMD 0.32 lower (0.71 lower to 0.07 higher)		237 (4 studies)	⊕⊕⊕⊝ **moderate**^2^	As a rule of thumb, 0.2 SMD represents a small difference, 0.5 moderate, and 0.8 large.
Depression						
**Acupuncture vs. pharmacotherapy**		SMD 0.64 lower (0.94 to 0.35 lower)		1641 (18 studies)	⊕⊕⊝⊝ **low** ^3^^,^^4^^,^^5^	As a rule of thumb, 0.2 SMD represents a small difference, 0.5 moderate, and 0.8 large.
Anxiety						
**Acupuncture vs.pharmacotherapy**		SMD 0.46 lower (0.69 lower to 0.22 lower)		1743 (19 studies)	⊕⊕⊝⊝ **low** ^3^^,^^4^^,^^6^	As a rule of thumb, 0.2 SMD represents a small difference, 0.5 moderate, and 0.8 large.
Depression						
**Acupuncture vs. pharmacotherapy**	**Study population**		**RR 0.56**	847	⊕⊕⊕⊝	
			(0.26 to 1.19)	(5 studies)	**moderate** ^3^	
Adverse events	**58 per 1000**	32 per 1000 (15 to 69)				

*The basis for the **assumed risk** (e.g. the median control group risk across studies) is provided in footnotes. The **corresponding risk** (and its 95% confidence interval) is based on the assumed risk in the comparison group and the **relative effect** of the intervention (and its 95% CI).

**CI**: Confidence interval; RR: Risk ratio; SMD: standard mean difference

GRADE Working Group grades of evidence

**High quality**: Further research is very unlikely to change our confidence in the estimate of effect.

**Moderate quality**: Further research is likely to have an important impact on our confidence in the estimate of effect and may change the estimate.

**Low quality**: Further research is very likely to have an important impact on our confidence in the estimate of effect and is likely to change the estimate.

**Very low quality**: We are very uncertain about the estimate.

^1^ Substantial heterogeneity (I2 = 85%, P<0.01)

^2^ The sample size of each group is less than 200

^3^ High risk of performance and detection bias owing to nonblinding.

^4^ Trim-and-fill analysis was used to prove that the conclusion will not be affected by publication bias.

^5^ Substantial heterogeneity (I2 = 86%, P<0.01)

^6^ Substantial heterogeneity (I2 = 79%, P<0.01)

#### 3.4.2 Depression

The pooled data showed that acupuncture was not significantly better at relieving depression symptoms than sham acupuncture (SMD -0.32, 95% CI −0.71 to 0.07, I^2^ = 54%, Fig 2B), and we also constructed a drapery plot (S7 Fig in S1 File) to visualize the results. The GRADE quality of the evidence was moderate (Table 2). The sensitivity analyses showed that acupuncture reduced depression symptoms more than the control when the study Da2014 was omitted (SMD -0.44, 95% CI -0.84 to -0.03, S8 Fig in S1 File), but the effect was very small. Moreover, the subgroup analysis based on disease showed that acupuncture may not be superior in relieving depression symptoms in patients with IBS, FD, FC and FDr compared with sham acupuncture (Fig 2B). The subgroup analysis based on acupoints showed that acupuncture prescriptions include acupoints for tranquilization, may be more useful in relieving depression symptoms in patients with FGIDs than sham acupuncture (S9 Fig in S1 File). The subgroup analysis based on type of acupuncture showed that no matter electroacupuncture or manual acupuncture could relieve depression symptoms in patients with FGIDs to a greater degree than the control (S8 Fig in S1 File).

### 3.5 Acupuncture versus pharmacotherapy

#### 3.5.1 Anxiety

**Fig 3:**
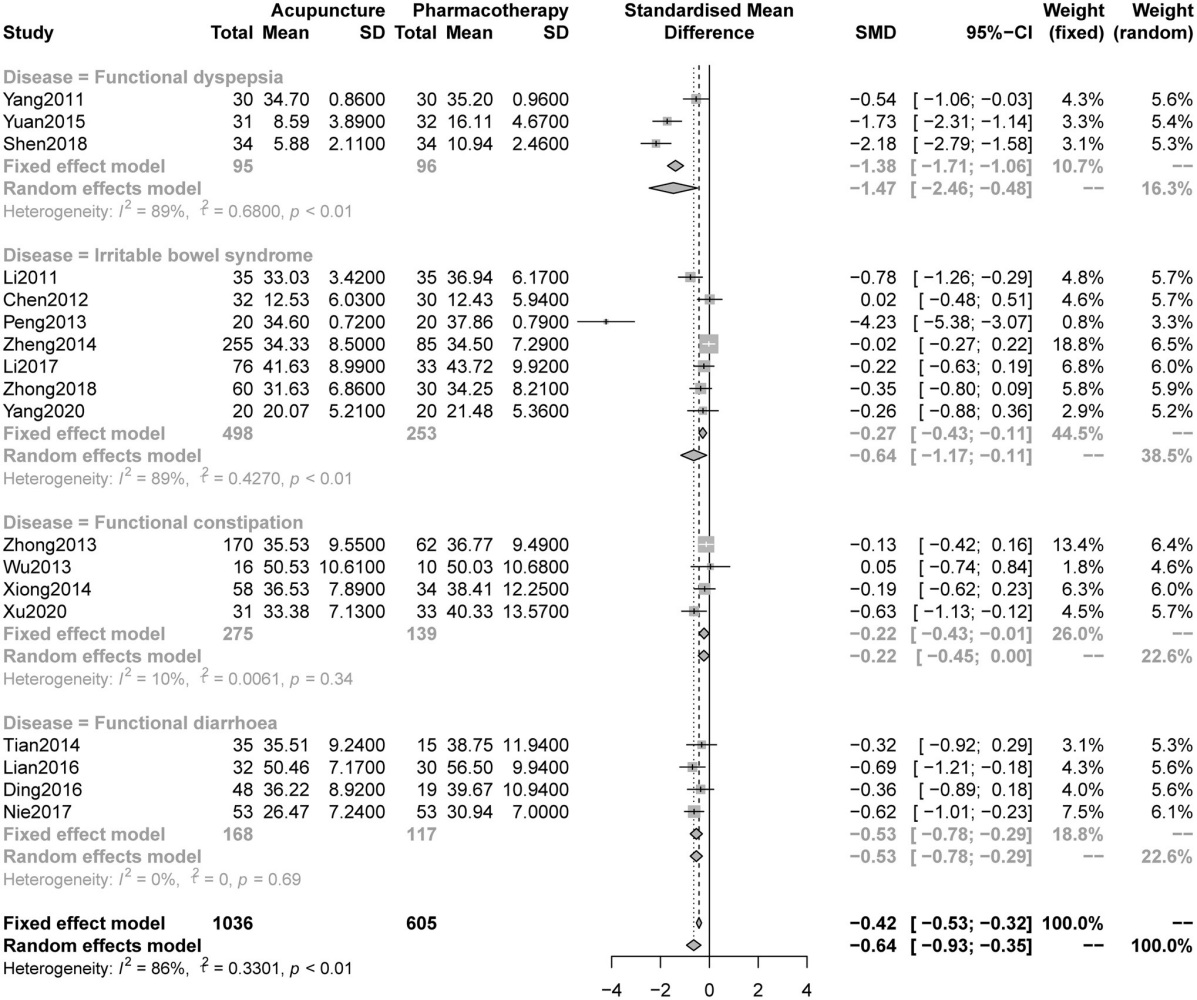
Forest plot of comparison: Acupuncture versus pharmacotherapy, outcome: Severity of anxiety at the end of treatment.

The pooled results indicated that acupuncture was significantly better at relieving anxiety symptoms than pharmacotherapy (SMD -0.64, 95% CI -0.93 to -0.35, I2 = 86%, Fig 3). A drapery plot was constructed to visualize the results (S11 Fig in S1 File). The GRADE quality of the evidence was low (Table 2). The sensitivity analyses confirmed the robustness of the results (S12 Fig in S1 File). Moreover, the subgroup analysis (Fig 3) showed that acupuncture was better than pharmacotherapy at relieving anxiety symptoms in patients with FD (SMD -1.47, 95% CI -2.46 to -0.48), IBS (SMD -0.64, 95% CI -1.17 to -0.11), FC (SMD -0.22, 95%CI -0.43 to -0.01) and FDr (SMD -0.53, 95% CI -0.78 to -0.29). The results of the other subgroup analyses showed that this effect was independent of the inclusion of tranquilization acupoints (S13 Fig in S1 File) and the type of acupuncture (S14 Fig in S1 File).

#### 3.5.2 Depression

**Fig 4:**
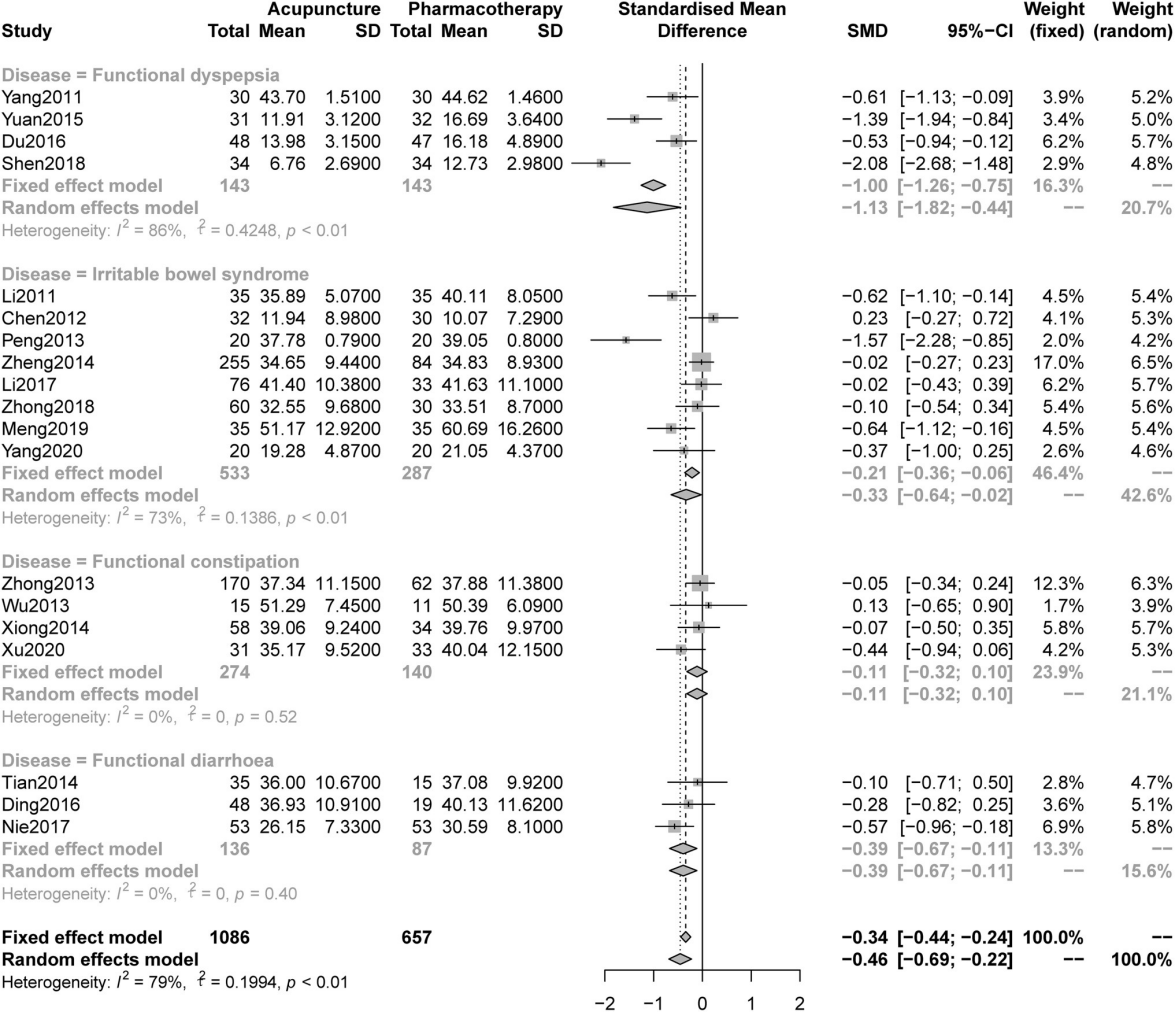
Forest plot of comparison: Acupuncture versus pharmacotherapy, outcome: Severity of depression at the end of treatment.

The analysis of pooled data indicated that acupuncture was significantly better at improving depression symptoms than pharmacotherapy (SMD -0.46, 95% CI -0.69 to -0.22, I^2^ = 79%, Fig 4); we constructed a drapery plot to visualize the results (S15 Fig in S1 File). The overall quality of the evidence was low (Table 2). The sensitivity analyses confirmed the robustness of the results (S16 Fig in S1 File). In addition, the subgroup analysis (Fig 3) showed that acupuncture was better than pharmacotherapy at relieving anxiety symptoms in patients with FD (SMD -1.13, 95% CI -1.82 to -0.44), IBS (SMD -0.33, 95% CI -0.64 to -0.02) and FDr (SMD -0.39, 95% CI -0.67 to -0.11), but not FC (SMD -0.11, 95%CI -0.32 to 0.10). The other subgroup analyses indicated that this effect had nothing to do with the inclusion of tranquilization acupoints (S17 Fig in S1 File) or acupuncture type (S18 Fig in S1 File).

### 3.6 Adverse events

**Fig 5:**
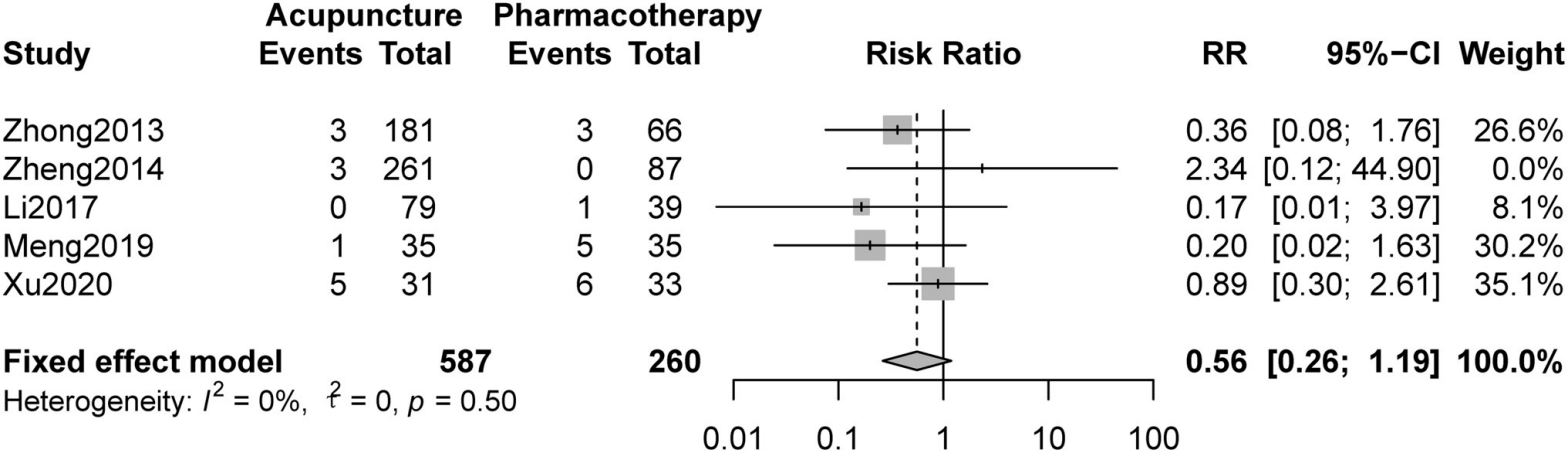
Forest plot of comparison: Acupuncture versus pharmacotherapy, outcome: Adverse events.

Nine studies reported adverse events, and 4 of them reported 0 adverse events in all groups. No serious adverse events occurred in the 9 studies. Five studies compared acupuncture and pharmacotherapy and reported the number of adverse events. The pooled result indicated that there were no statistically significant differences between the groups (RR 0.56, 95%CI 0.26 to 1.19, Fig 5).

### 3.7 Publication bias

The contour-enhanced funnel plots (S19 Fig in S1 File) and Egger’s test (P = 0.004) for the effects of acupuncture versus pharmacotherapy on anxiety suggested possible publication bias. After adding 1 studies based on the Duval & Tweedie trim-and-fill method [40], the SMD was statistically significant both before and after trim-and-fill analysis. Moreover, the contour-enhanced funnel plots (S20 Fig in S1 File) and Egger’s test (P = 0.027) for the effects of acupuncture versus pharmacotherapy on depression showed potential publication bias. The trim-and-fill method was used to add 2 studies, and the SMD was statistically significant as before trim-and-fill analysis.

## 4. Discussion

This meta-analysis included 2151 patients from 24 RCTs. We compared acupuncture versus sham acupuncture and pharmacotherapy. The pooled results showed that acupuncture was relatively effective in relieving anxiety and depression symptoms in patients with FGIDs, but the current evidence does not explain whether this effect is a placebo effect. However, heterogeneity and high risk of bias reduced our level of certainty regarding this evidence.

FGIDs are currently defined as gut-brain interaction disorders, and the associated gastrointestinal symptoms may lead to emotional symptoms, such as anxiety and depression, which can aggravate gastrointestinal symptoms, resulting in a vicious cycle. Therefore, a treatment that can relieve both gastrointestinal symptoms and psychological symptoms in patients with FGIDs will be more beneficial in controlling such symptoms. Acupuncture has been used in China for thousands of years. In recent years, studies have found that acupuncture can be used to treat gastrointestinal diseases as well as emotional symptoms. However, the acupoints used in acupuncture for the treatment of different diseases are often quite different. For example, Tianshu, Zusanli, Quchi, Shangjuxu and other specific acupoints related to the stomach are often used in the treatment of gastrointestinal diseases. Baihui, Yintang, Shenmen and other acupoints for tranquillization are often used in the treatment of emotional symptoms. According to traditional acupuncture theory, it is believed that the functions of acupoints include distal treatment, near treatment and special treatment. Distal-treatment function means that acupoints can treat diseases where the meridians pass, near-treatment function means that acupoints can treat diseases where they are located, and special-treatment function means that some acupoints have a specific effect on some diseases. These effects are called specific effects in clinical practice. In addition, modern studies believe that acupuncture has nonspecific effects, including expectation effects, Hawthorne effects, Pygmalion effects and so on [41]. In the subgroup analysis, this study found that regardless of whether the acupuncture prescription included tranquilization acupoints, its efficacy in relieving psychological symptoms in patients with FGIDs was higher than that of conventional drug therapy, but the result was opposite when compared with sham acupuncture. This suggests that acupuncture to alleviate psychological symptoms in patients with FGIDs may be mostly due to its nonspecific effect, while the pseudoacupuncture method at nonacupoints has not only a placebo effect but also a nonspecific effect. These results can also be explained in terms of the mechanism. Acupuncture at Tianshu, Zusanli, and Shangjuxu can regulate the balance of the gut microbiome [42–45], and gut microbiome dysbiosis is an important cause of depression and anxiety [46, 47]. Therefore, acupuncture can also alleviate psychological symptoms that rely on brain-gut axis interactions when the prescription does not include tranquilization acupoints. The mechanism by which acupuncture regulates the brain-gut axis to treat emotional symptoms is a current research hotspot. Compared with pharmacotherapy, acupuncture reduced anxiety and depressive symptoms in patients with FD, IBS and FDr and alleviated anxiety symptoms in patients with FC, but there was no significant difference in the scores for depression symptoms in patients with FC. Four RCTs were included in the FC subgroup analysis. All the RCTs used mosapride as the control; mosapride has antidepressant and antianxiety effects at routine clinical dosages [48]. This showed that acupuncture treatment for depression and anxiety symptoms in patients with FC was at least equivalent to mosapride. In addition, this study found that the type of acupuncture was not related to the effect of acupuncture in the treatment of emotional symptoms in FDIG patients, and both manual acupuncture and electroacupuncture can reduce their psychological symptoms to a greater degree than conventional drugs. In terms of adverse reactions, acupuncture and conventional drugs for the treatment of FGIDs were associated with few adverse reactions, and there was no significant difference between the two. Previous studies have also shown that acupuncture is associated with fewer adverse reactions than antidepressants [8].

To the best of our knowledge, this is the first systematic review of acupuncture treatment for emotional symptoms in FGIDs patients. Our results show that acupuncture is beneficial to the improvement of psychological symptoms in patients with FGIDs, and previous studies have shown that acupuncture has an advantage in improving gastrointestinal symptoms in patients with FGIDs [7]. Therefore, this study provides new evidence supporting acupuncture as an alternative intervention scheme for FGIDs.

However, this study has some limitations. First, 23 of the 24 included studies were from mainland China, and 1 study was from Hong Kong, China. Second, only 2 multicentre studies, with a total sample size of more than 200 people, were included; the others were single-centre, small-sample studies. Third, because of the particularity of acupuncture therapy, blinding of subjects and intervention implementers cannot be performed like it can in drug studies, but random sequence generation, distribution concealment, and blinding of outcome evaluators and statistical analysts is feasible. However, there are still deficiencies in the design of these trials, which may lead to bias toward low-quality research methods. Therefore, the conclusions of this study need to be further verified.

## 5. Conclusion

This meta-analysis found that acupuncture can alleviate emotional symptoms in FGID patients better than pharmacotherapy. However, it is not clear whether this effect is based on the placebo effect, specific effect or nonspecific effect of acupuncture. The evidence should be proven by rigorously designed RCTs in the future.

## Supporting information

S1 File Supplementary S1-S22 Figs and S1, S2 Tables. (DOCX)
