# Supplementary material for: Acupuncture for emotional symptoms in patients with functional gastrointestinal disorders: A systematic review and meta-analysis
Source: PLoS One. 2022 Jan 27;17(1):e0263166. doi: 10.1371/journal.pone.0263166 (PMC8794137; doi:10.1371/journal.pone.0263166)
Supplement: S1 File — (DOCX) [file pone.0263166.s001.docx]

**Figure S1 Summary and risk of bias graph**

**
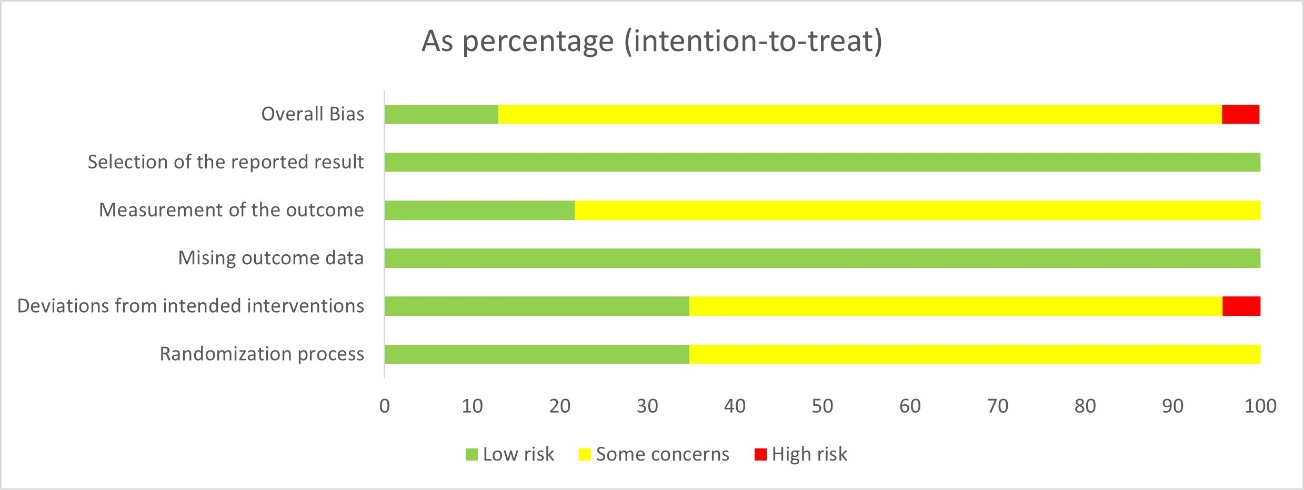
**

**Figure S2 Risk of bias figure**

**
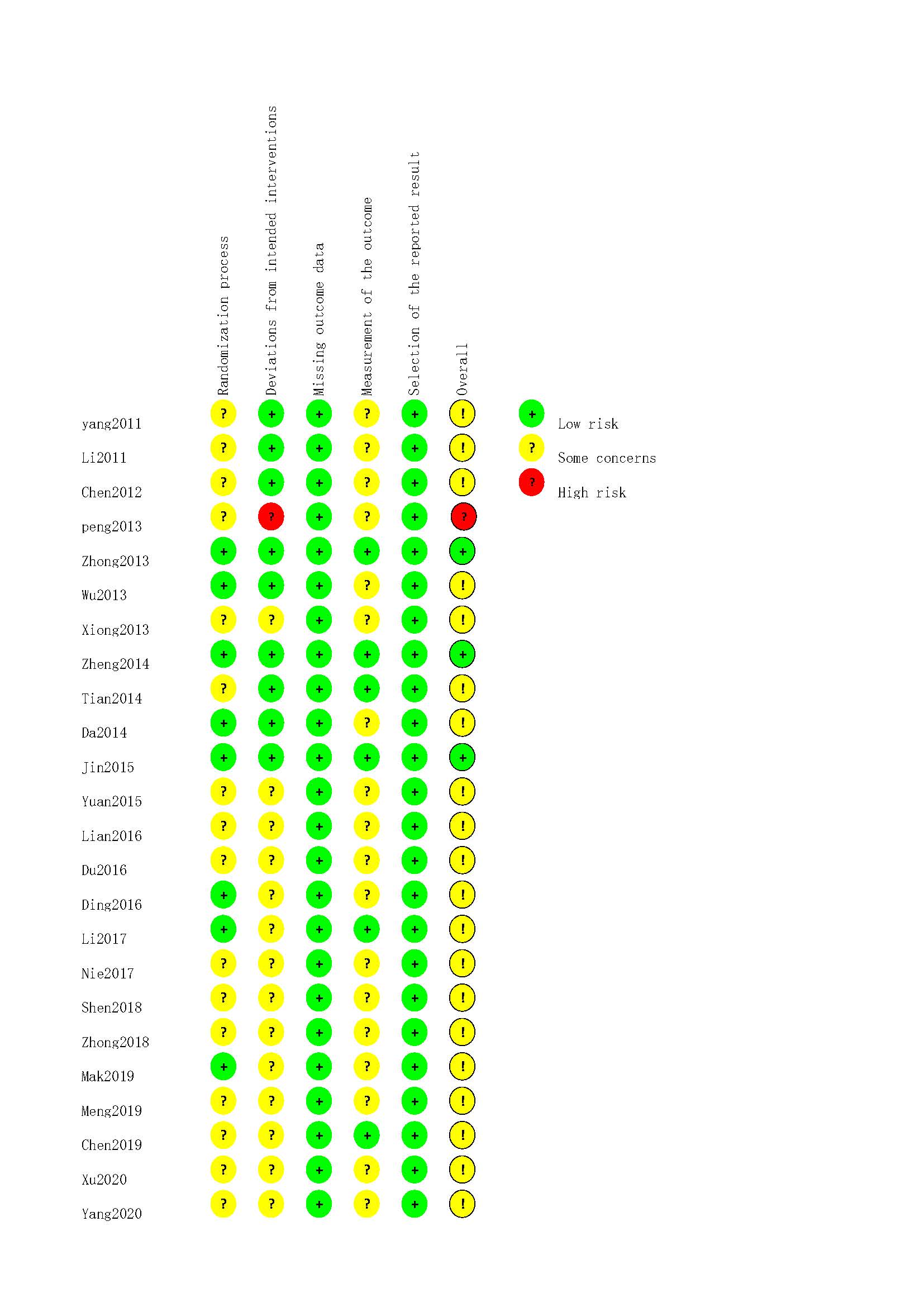
**

**Figure S3 Drapery plot of anxiety (Acupuncture VS Sham acupuncture)**

**
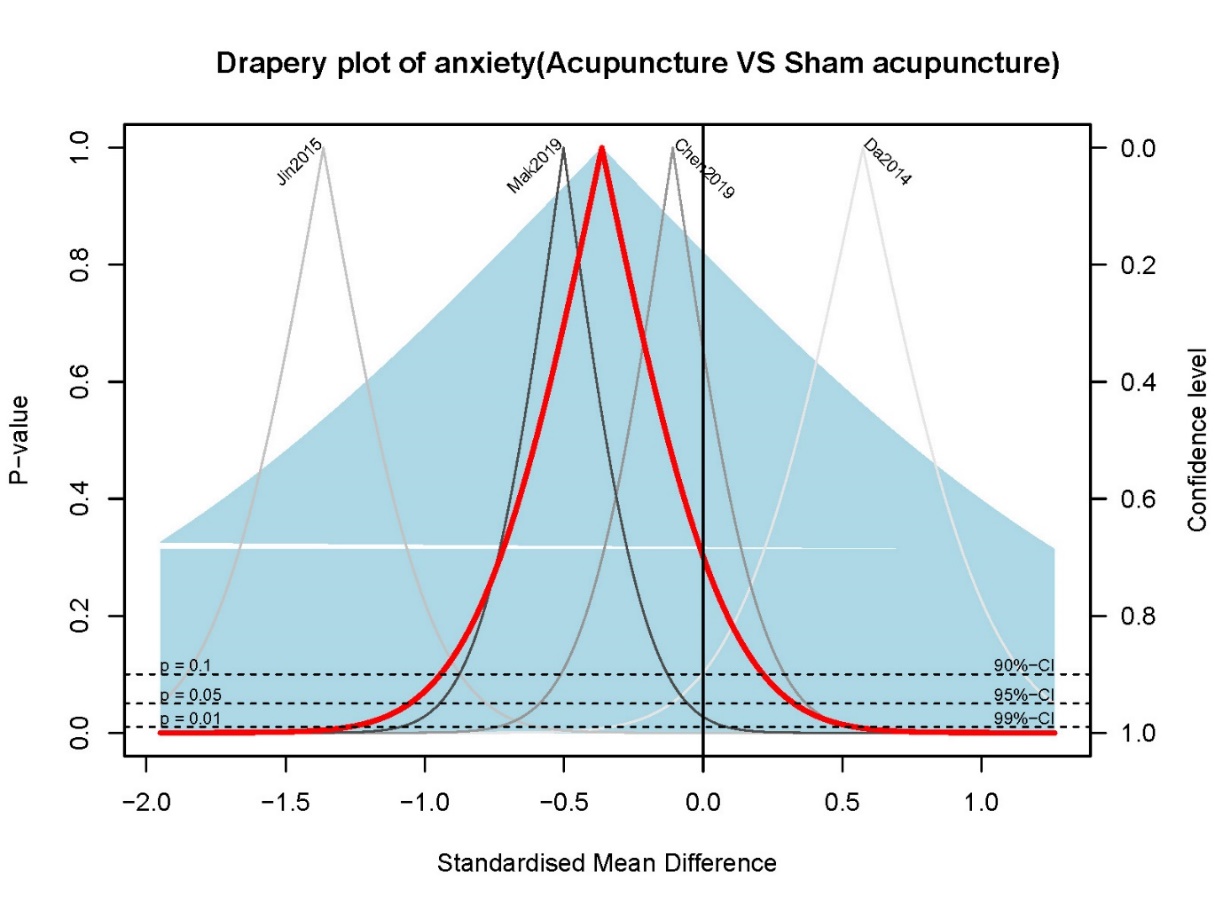
**

**Figure S4 Sensitivity analyses of anxiety (Acupuncture VS Sham acupuncture)**


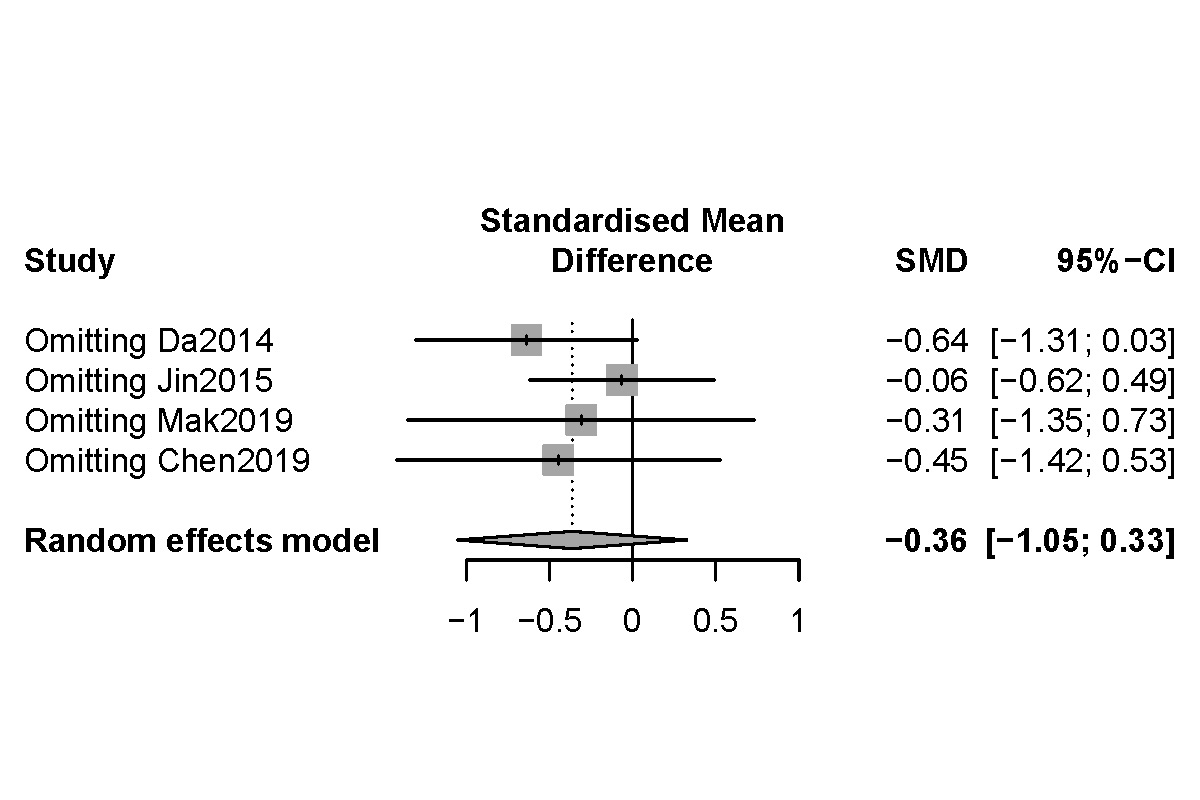


**Figure S5 Subgroup analyses of anxiety base on include acupoints for tranquillization or not (Acupuncture VS Sham acupuncture)**


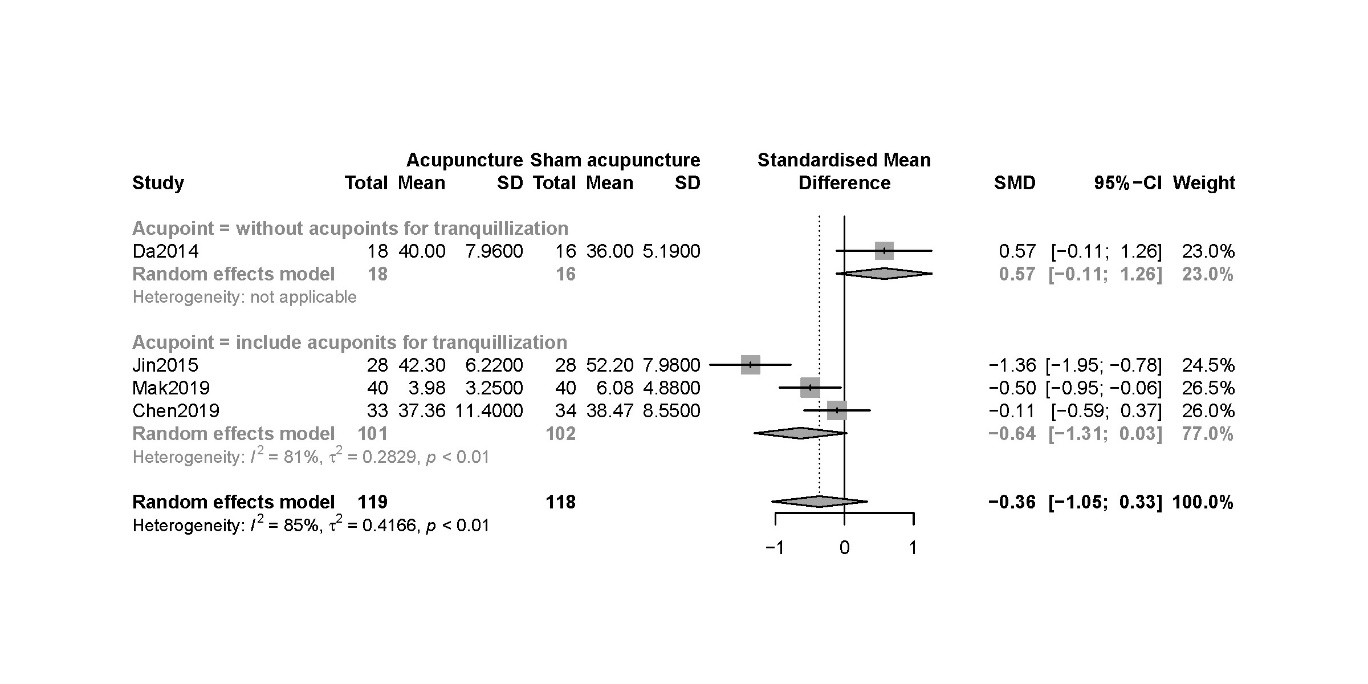


**Figure S6 Subgroup analyses of anxiety base on acupuncture type (Acupuncture VS Sham acupuncture)**


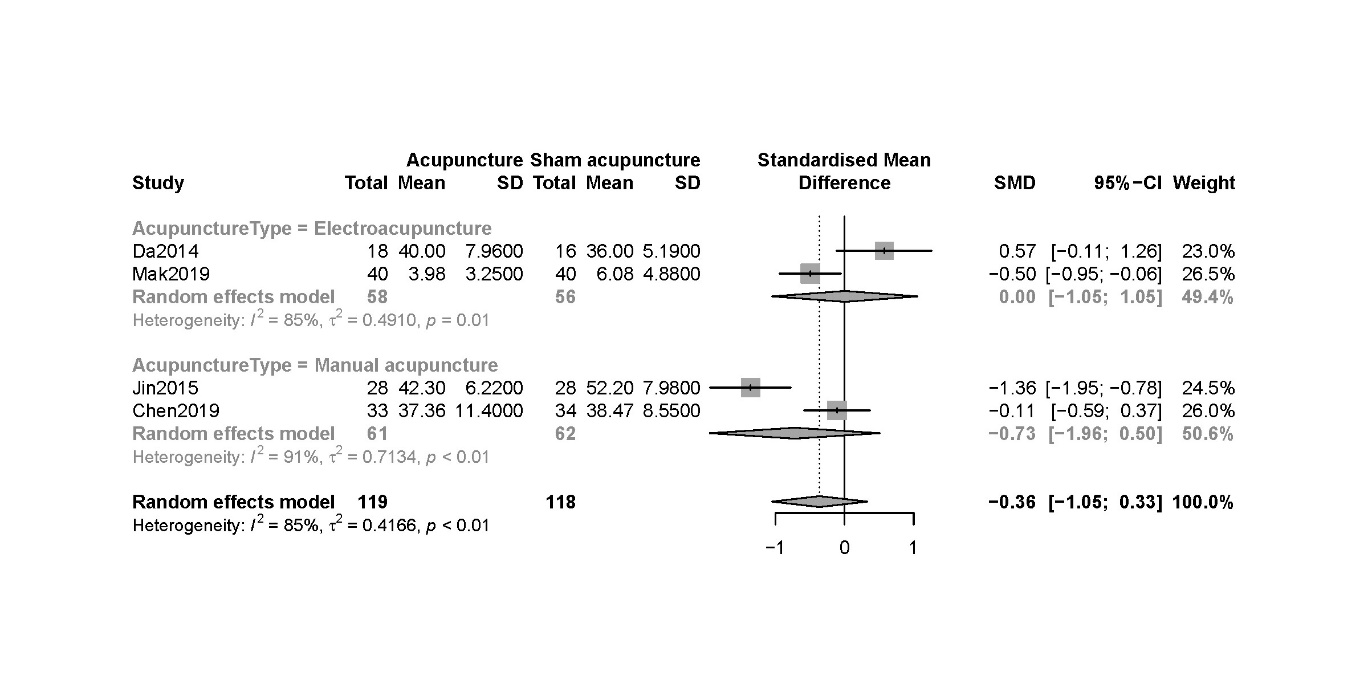


**Figure S7 Drapery plot of depression (Acupuncture VS Sham acupuncture)**


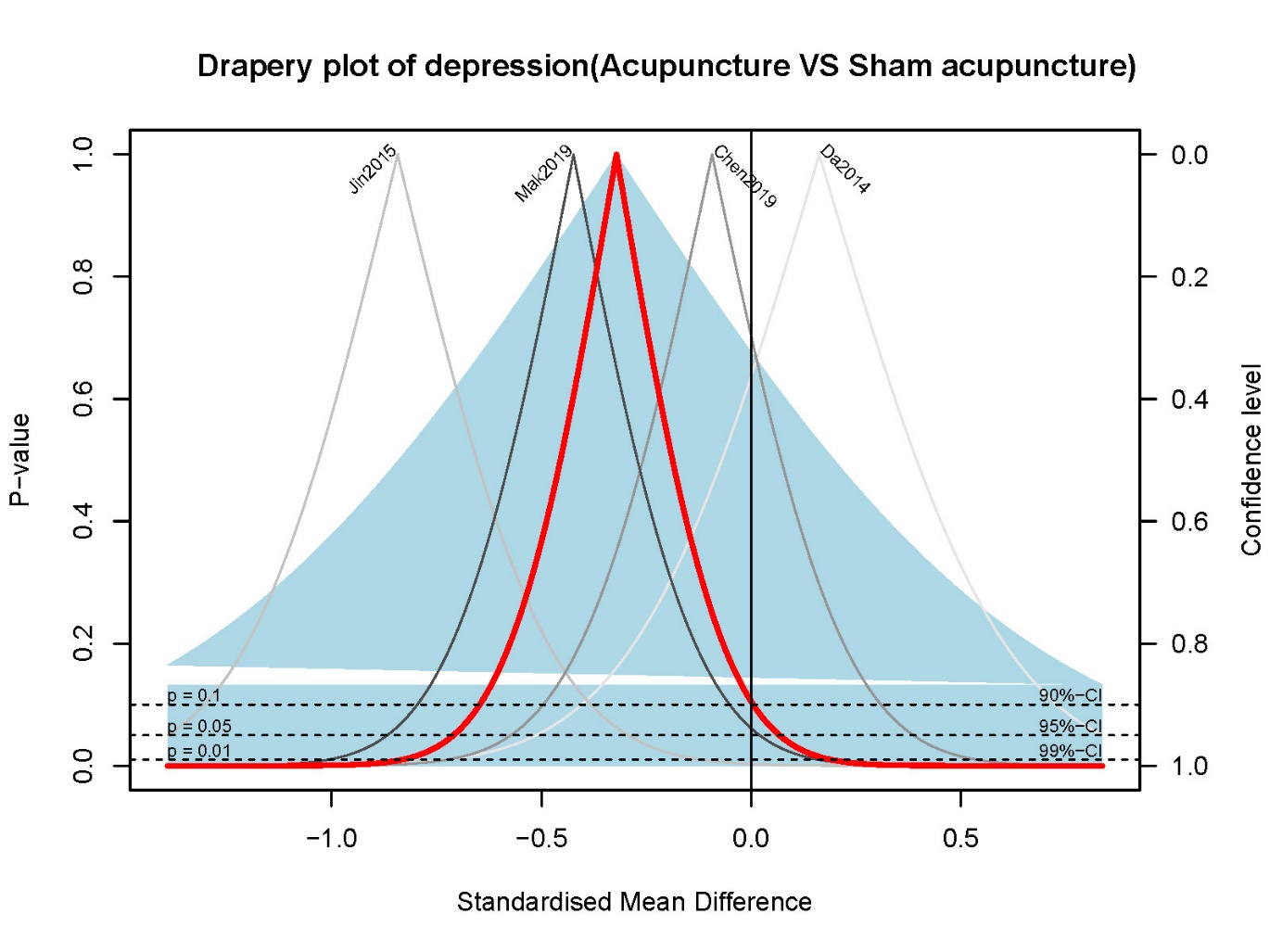


**Figure S8 Sensitivity analyses of depression (Acupuncture VS Sham acupuncture)**


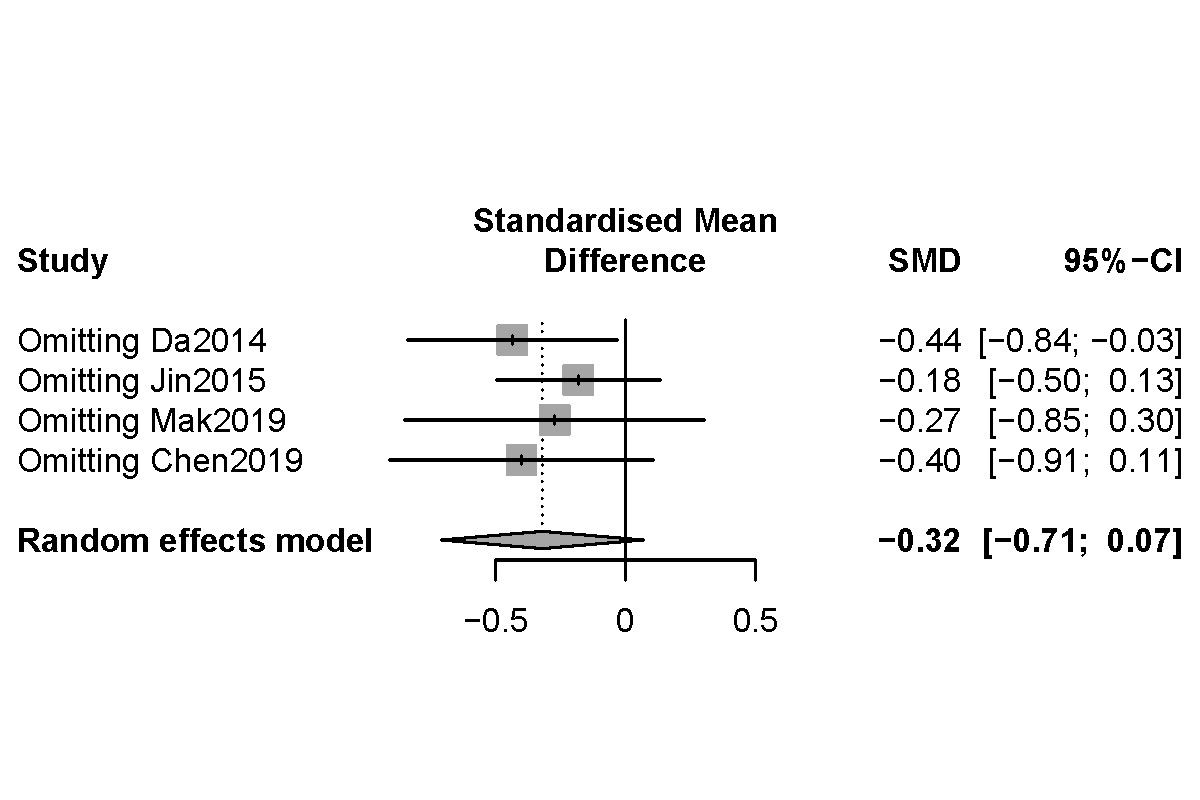


**Figure S9 Subgroup analyses of depression base on include acupoints for tranquillization or not (Acupuncture VS Sham acupuncture)**

**
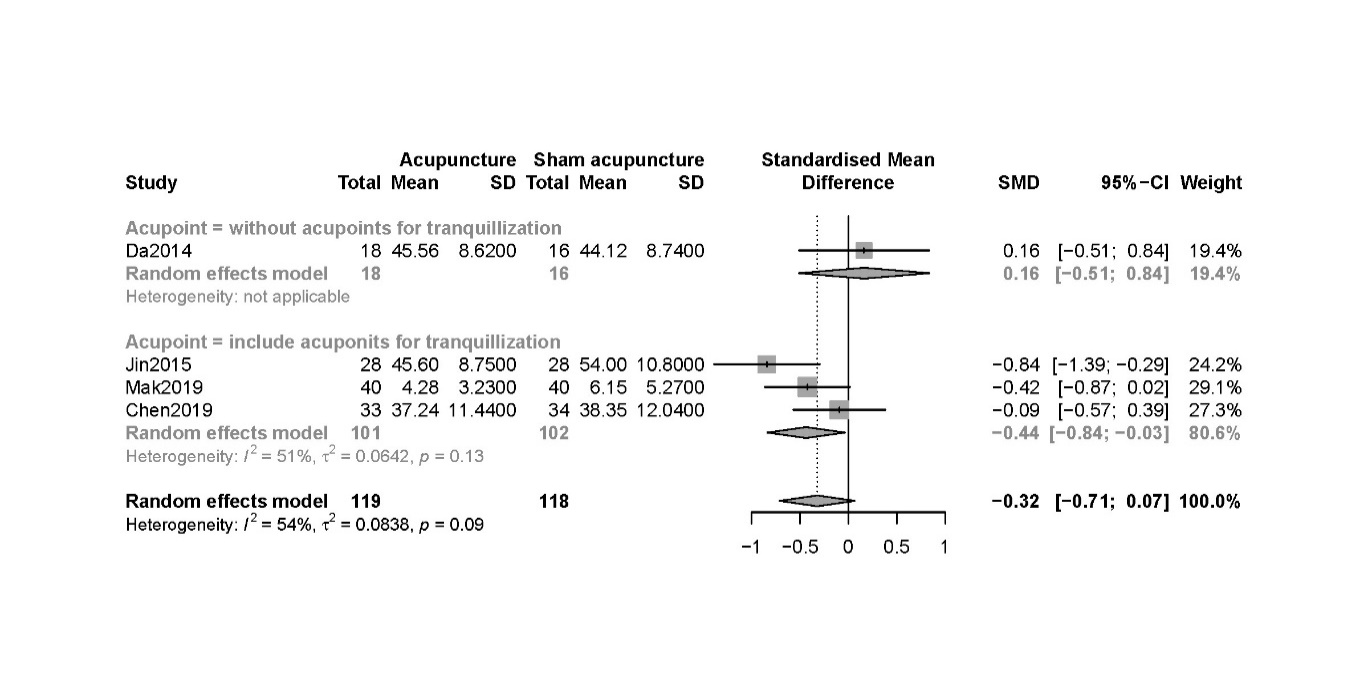
**

**Figure S10 Subgroup analyses of depression base on acupuncture type (Acupuncture VS Sham acupuncture)**


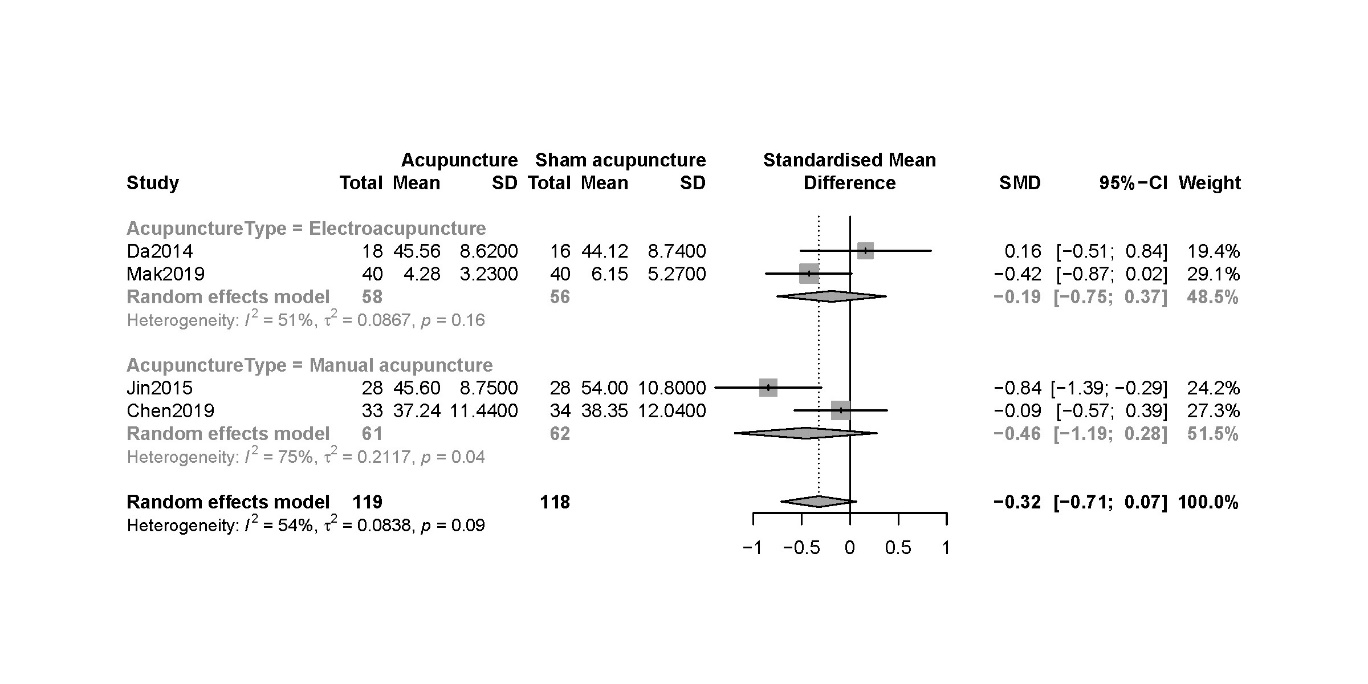


**Figure S11 Drapery plot of anxiety (Acupuncture VS Pharmacotherapy)**


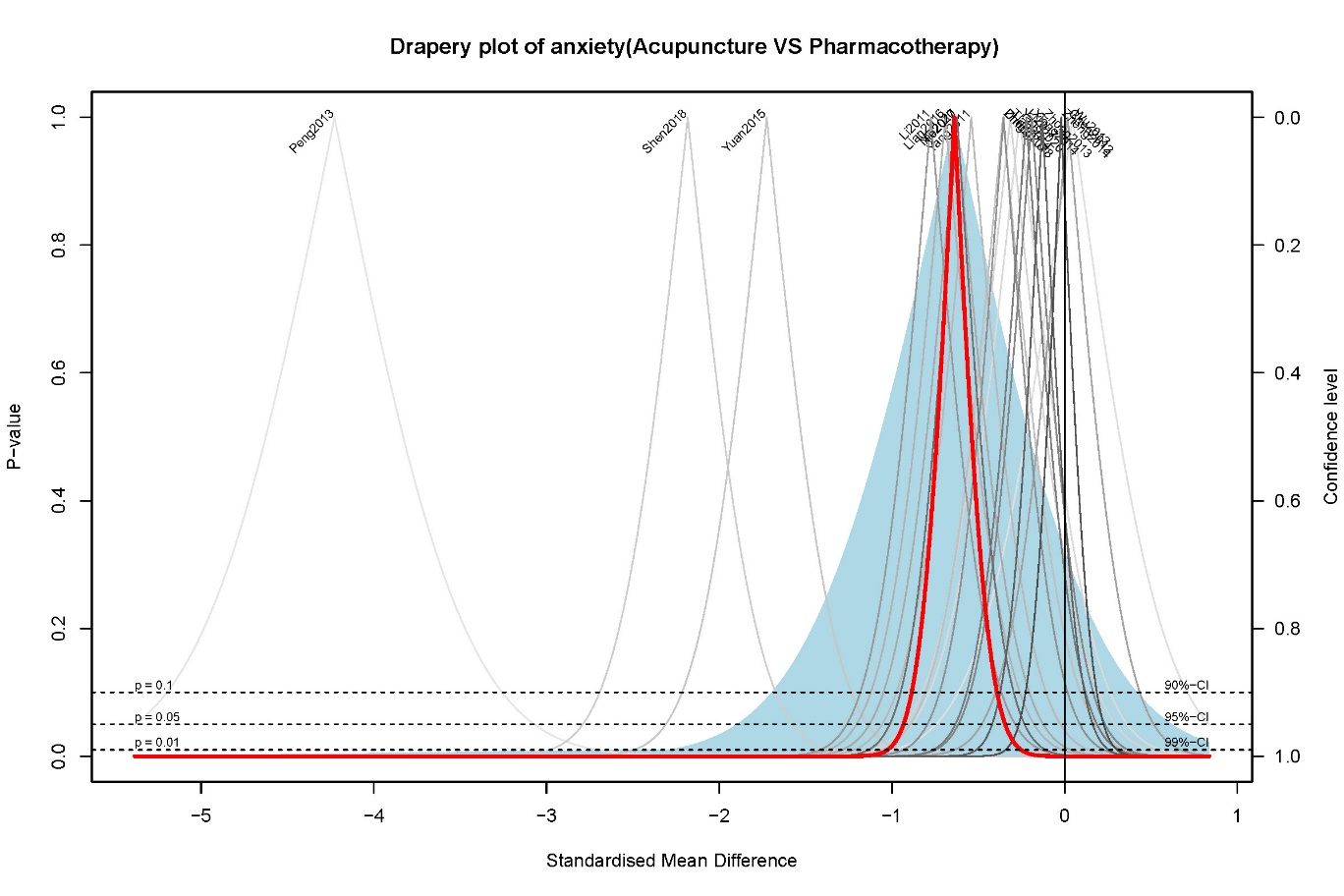


**Figure S12 Sensitivity analyses of anxiety (Acupuncture VS Pharmacotherapy)**


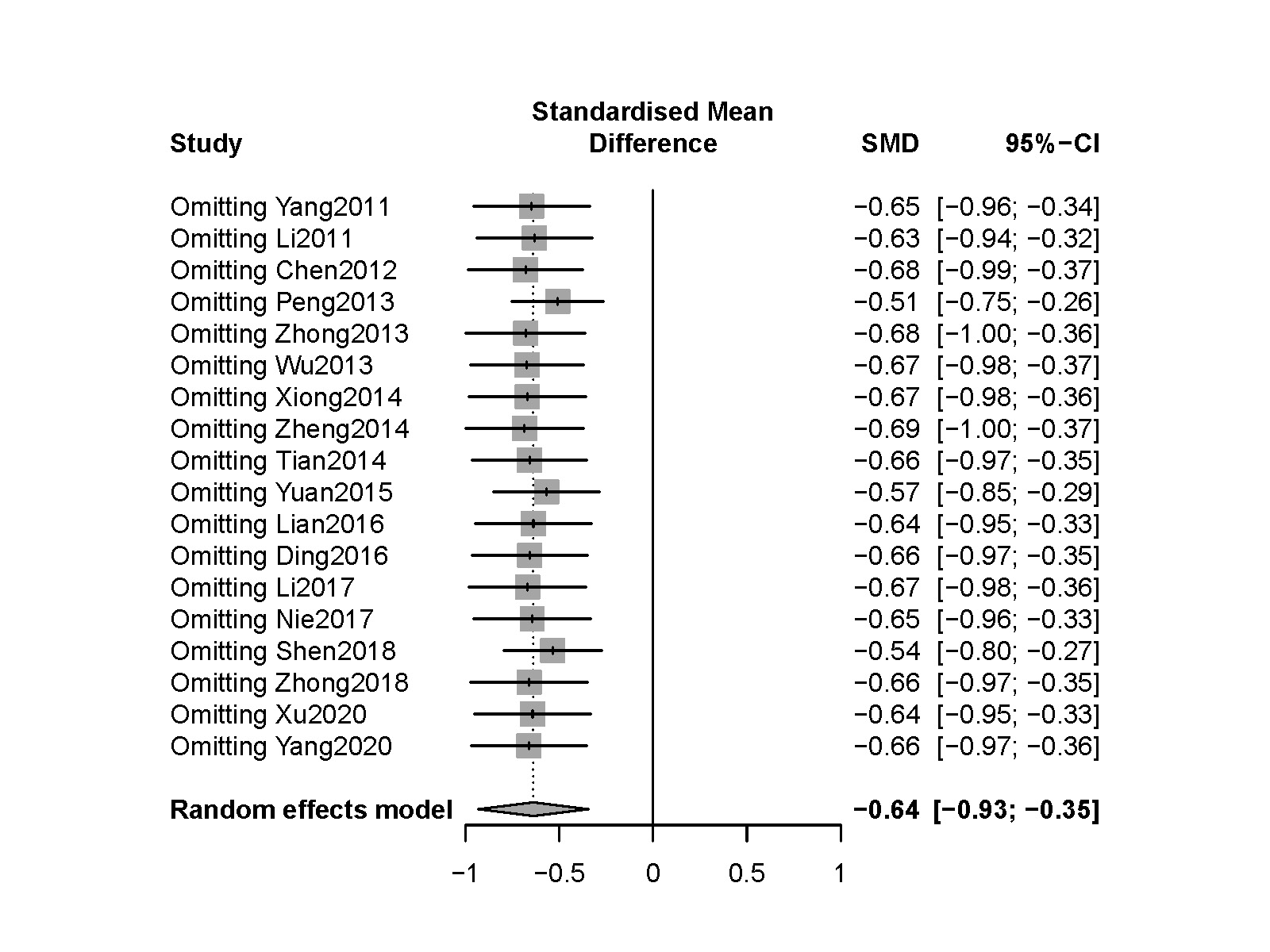


**Figure S13 Subgroup analyses of anxiety base on include acupoints for tranquillization or not (Acupuncture VS Pharmacotherapy)**


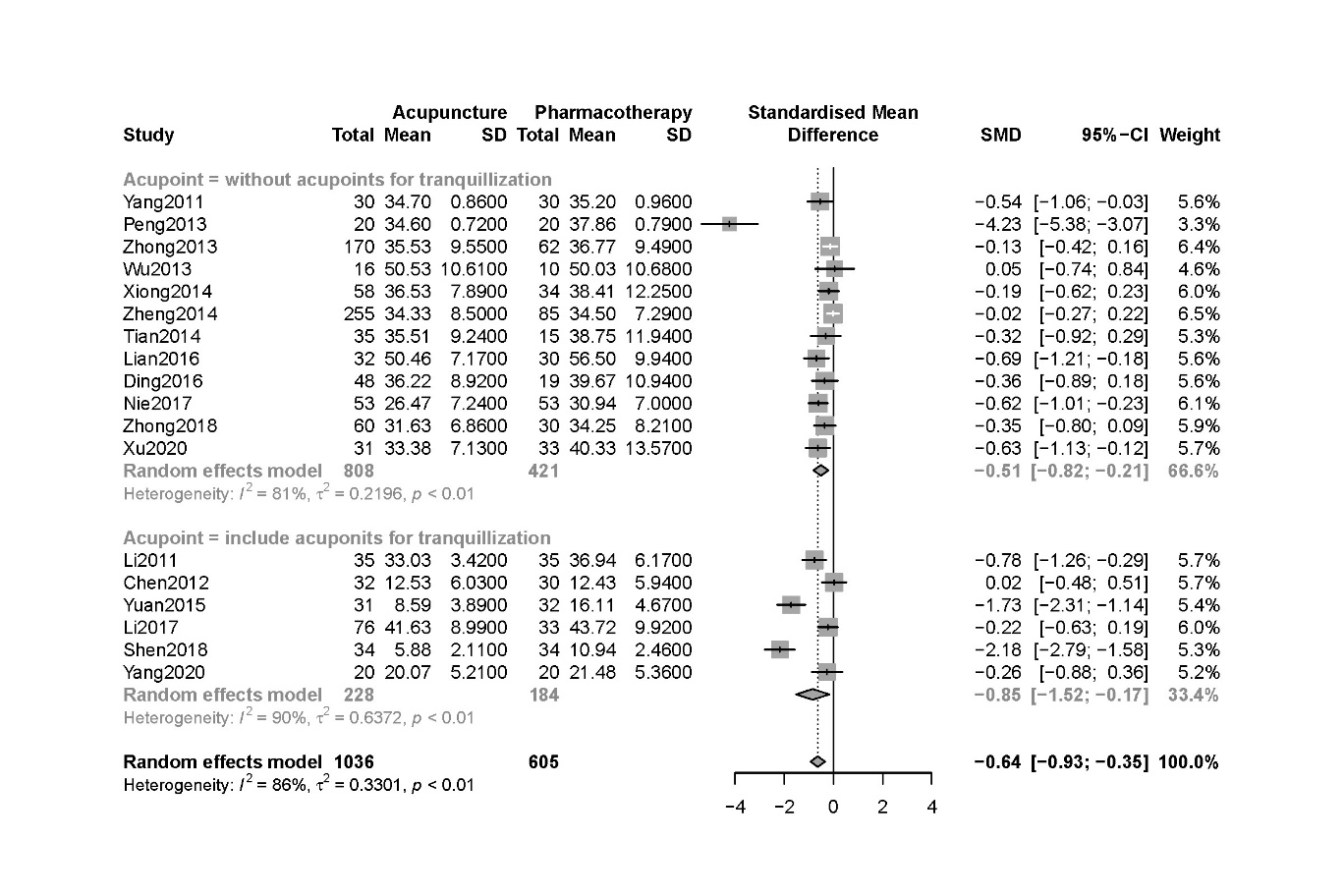


**Figure S14 Subgroup analyses of anxiety base on acupuncture type (Acupuncture VS Pharmacotherapy)**


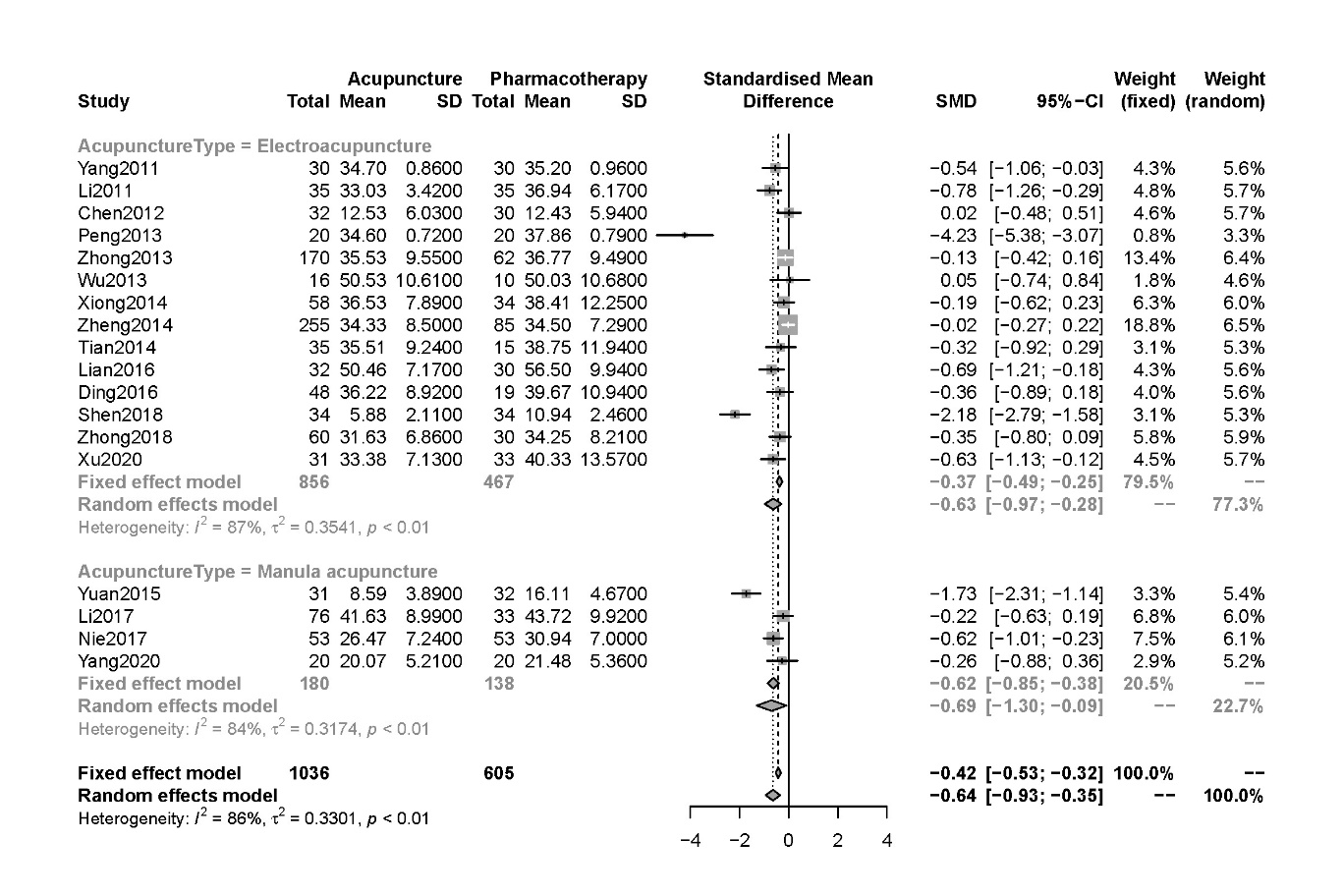


**Figure S15 Drapery plot of depression (Acupuncture VS Pharmacotherapy)**


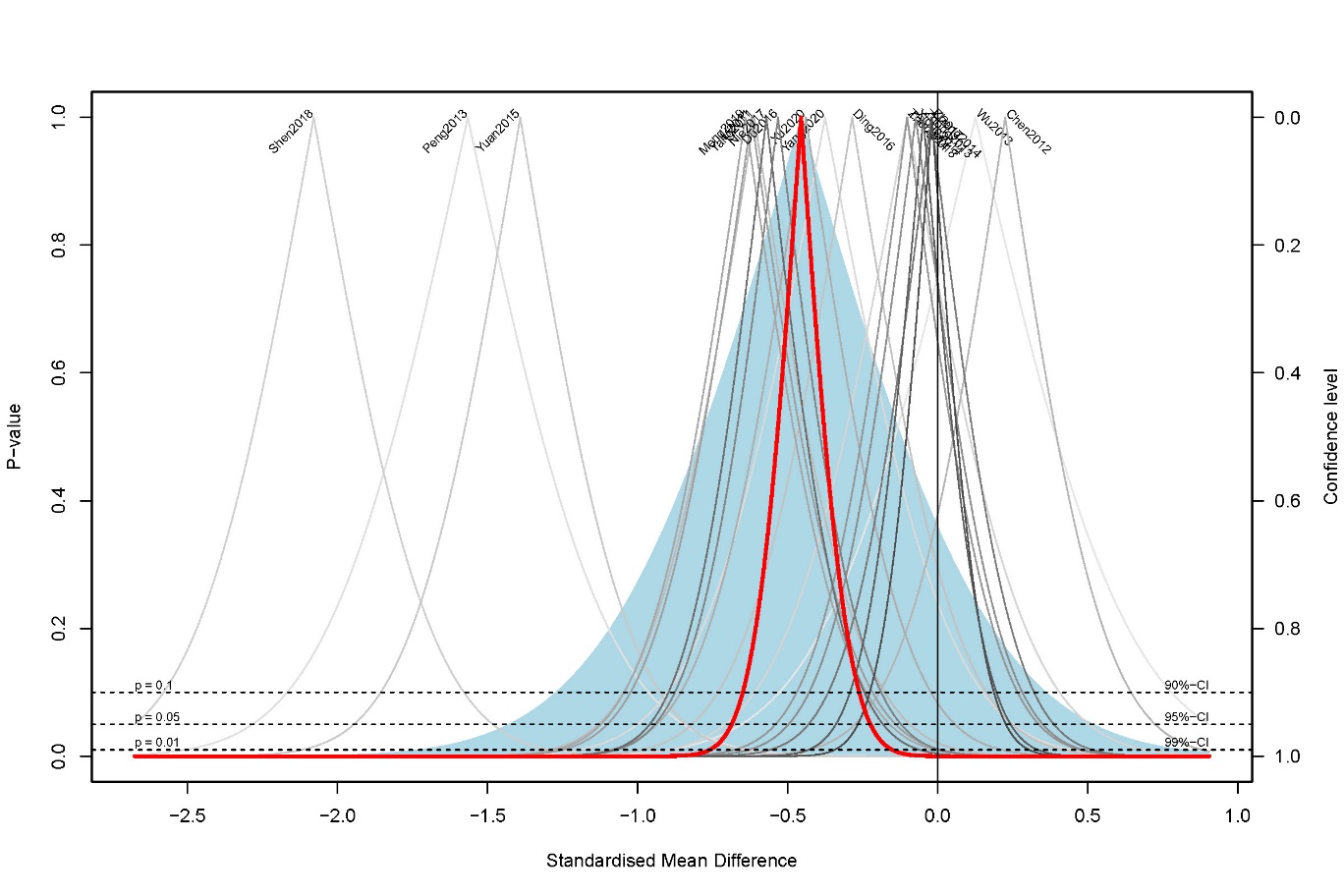


**Figure S16 Sensitivity analyses of depression (Acupuncture VS Pharmacotherapy)**


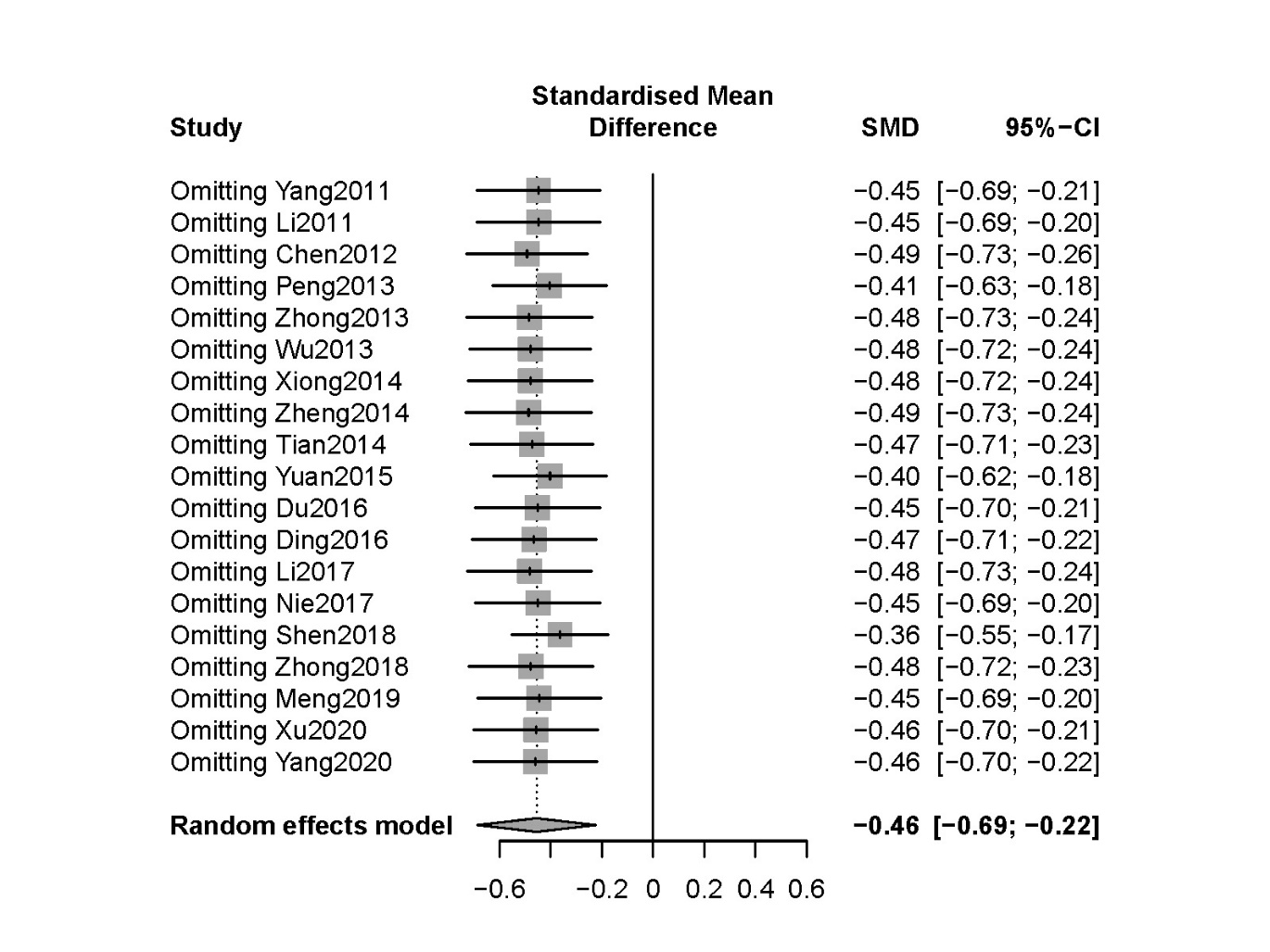
**Figure S17 Subgroup analyses of depression base on include acupoints for tranquillization or not (Acupuncture VS Pharmacotherapy)**


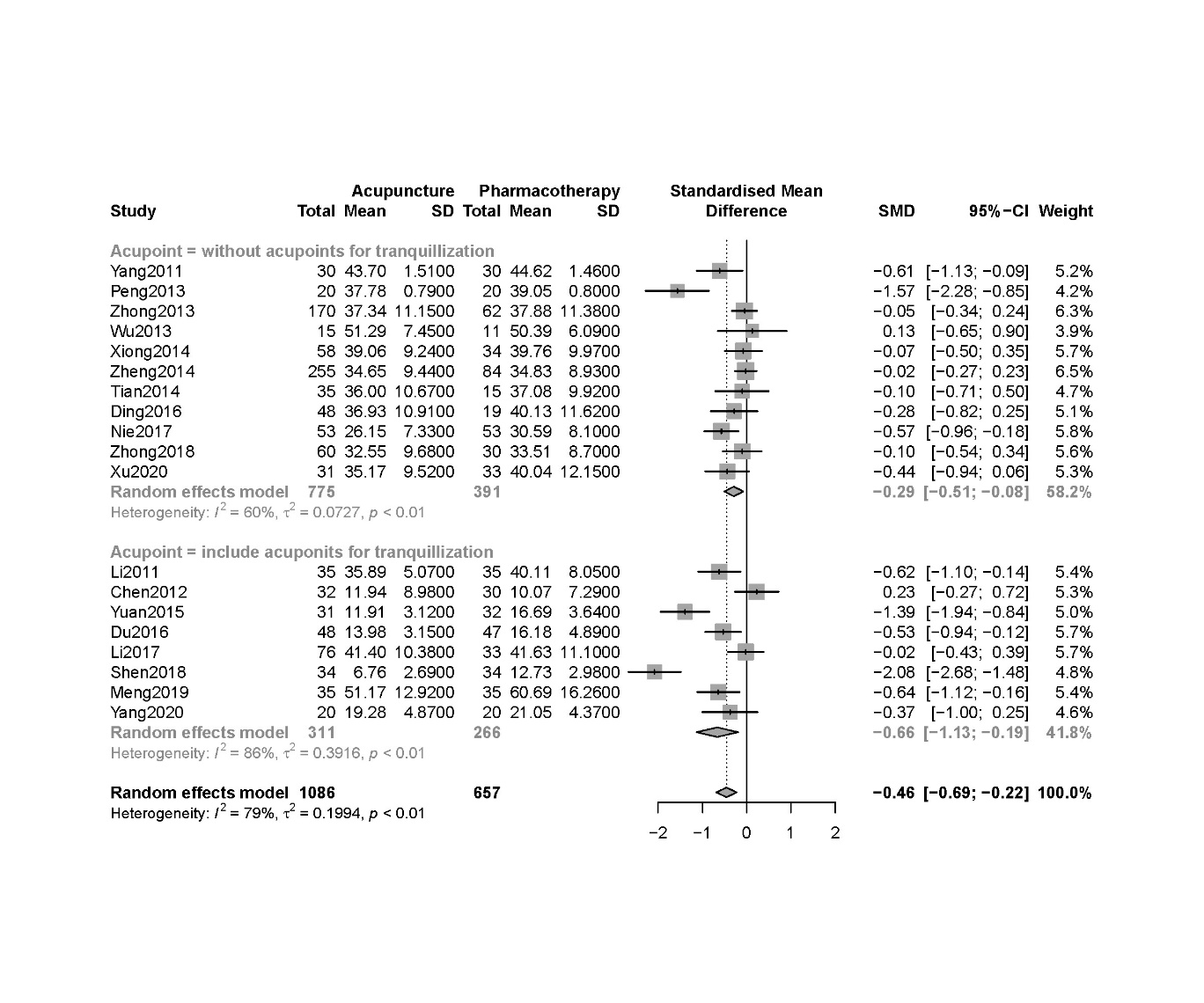


**Figure S18 Subgroup analyses of depression base on acupuncture type (Acupuncture VS Pharmacotherapy)**


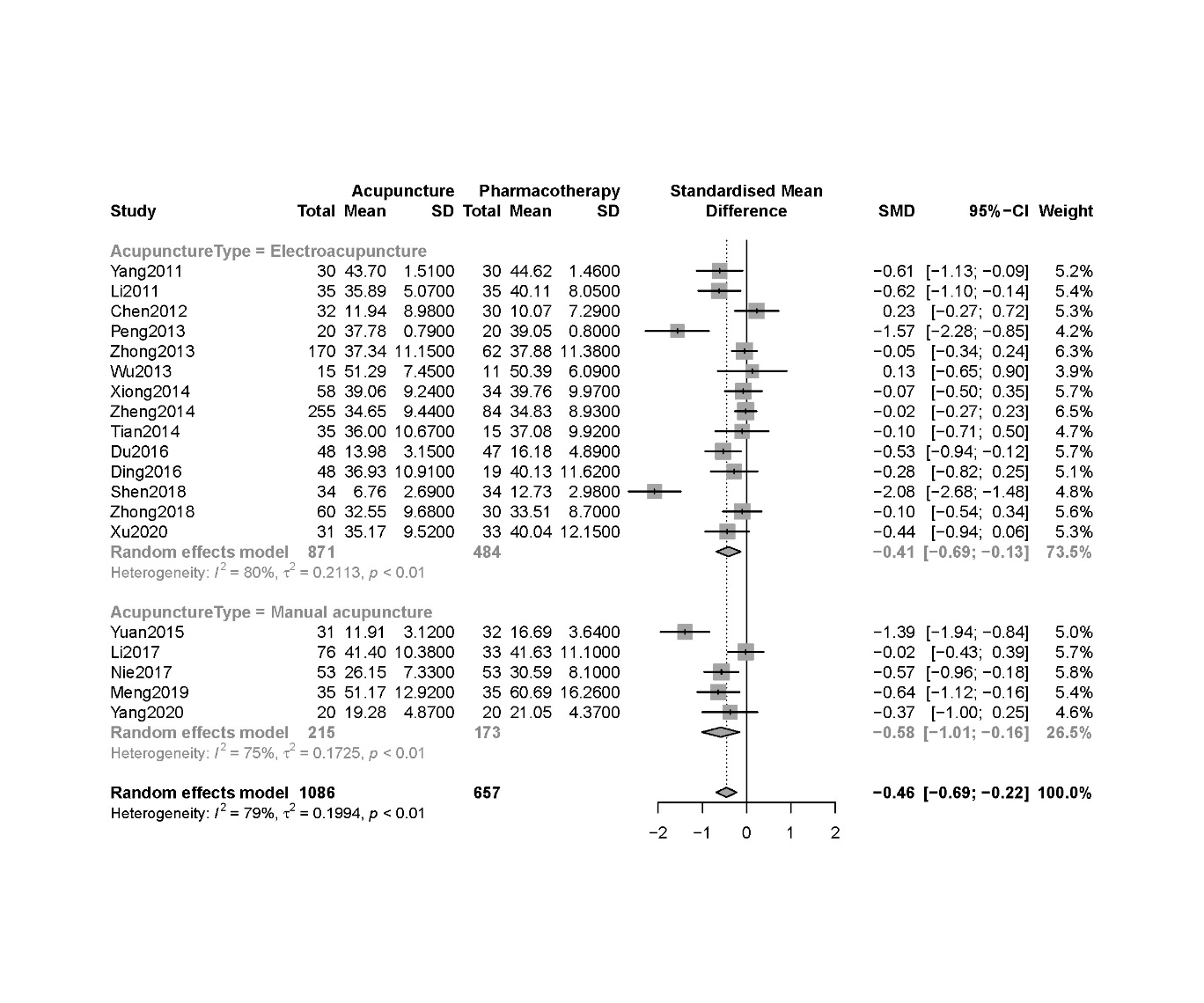


**Figure S19 Contour-enhanced funnel plot of anxiety (Acupuncture VS** **Pharmacotherapy)**

**
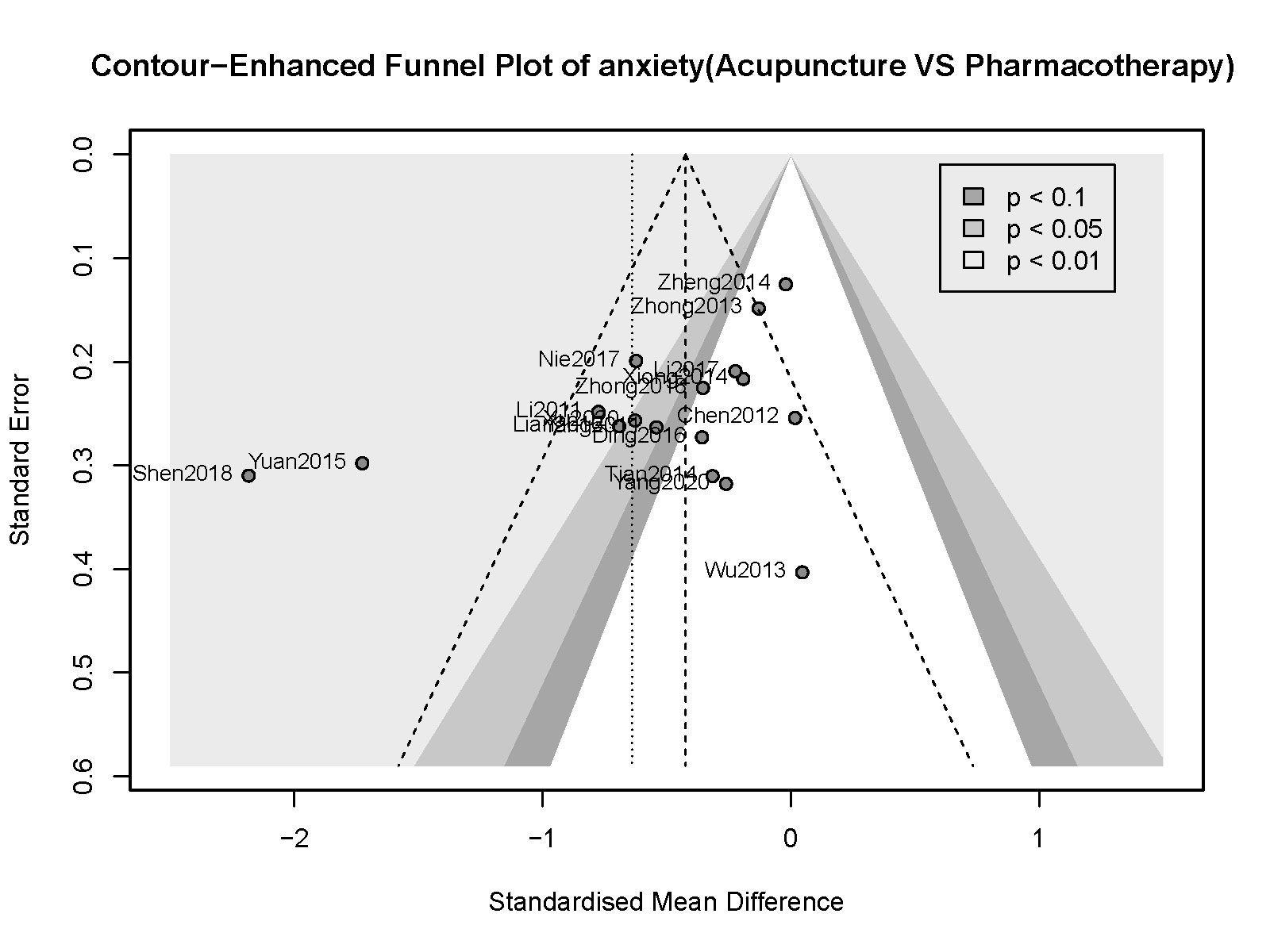
**

**Figure S20 Contour-enhanced funnel plot of depression (Acupuncture VS Pharmacotherapy)**

**
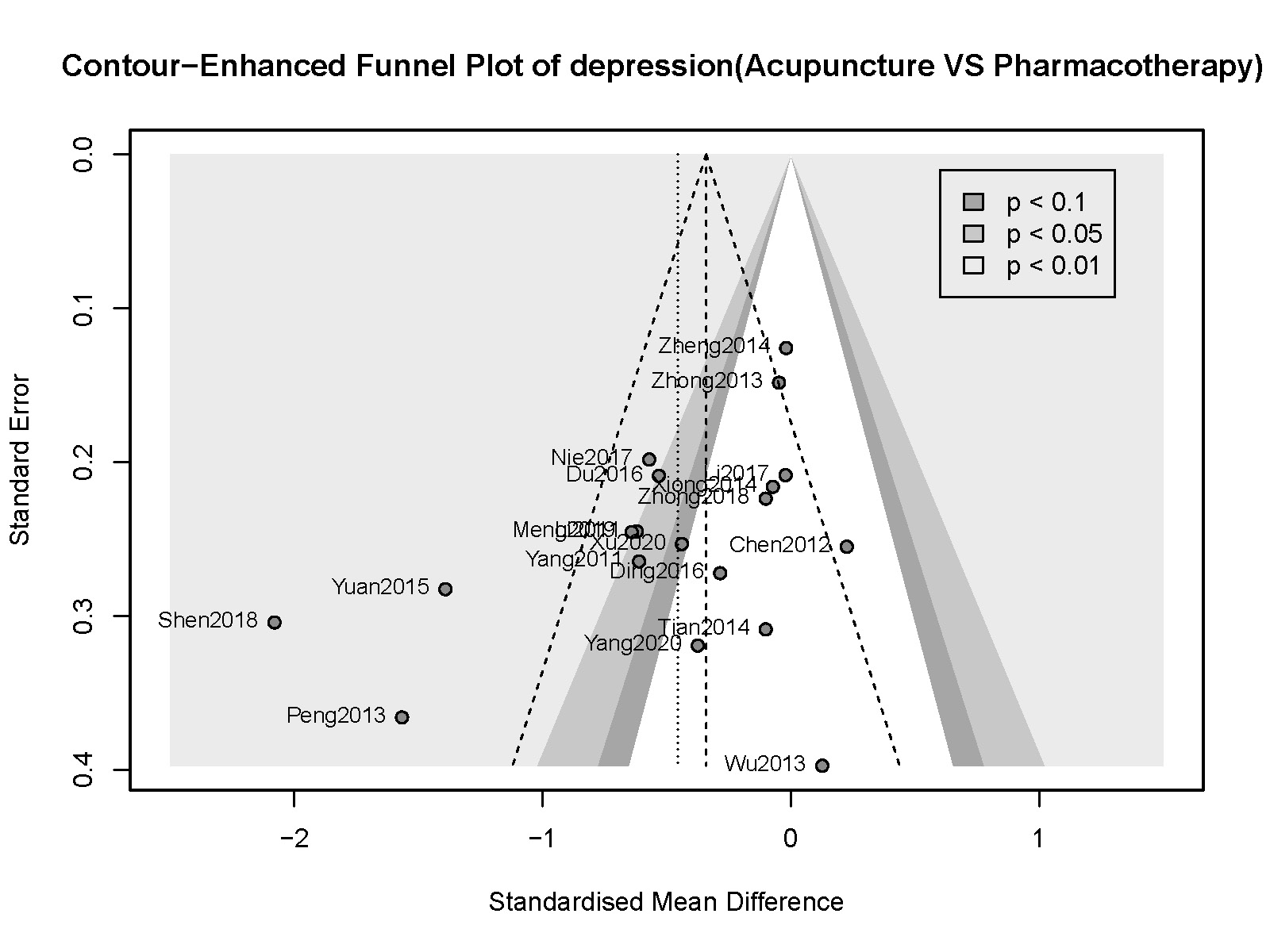
**

**Figure S21 Contour-enhanced funnel plot of anxiety (Acupuncture VS Pharmacotherapy) after trim-and-fill analysis**

**
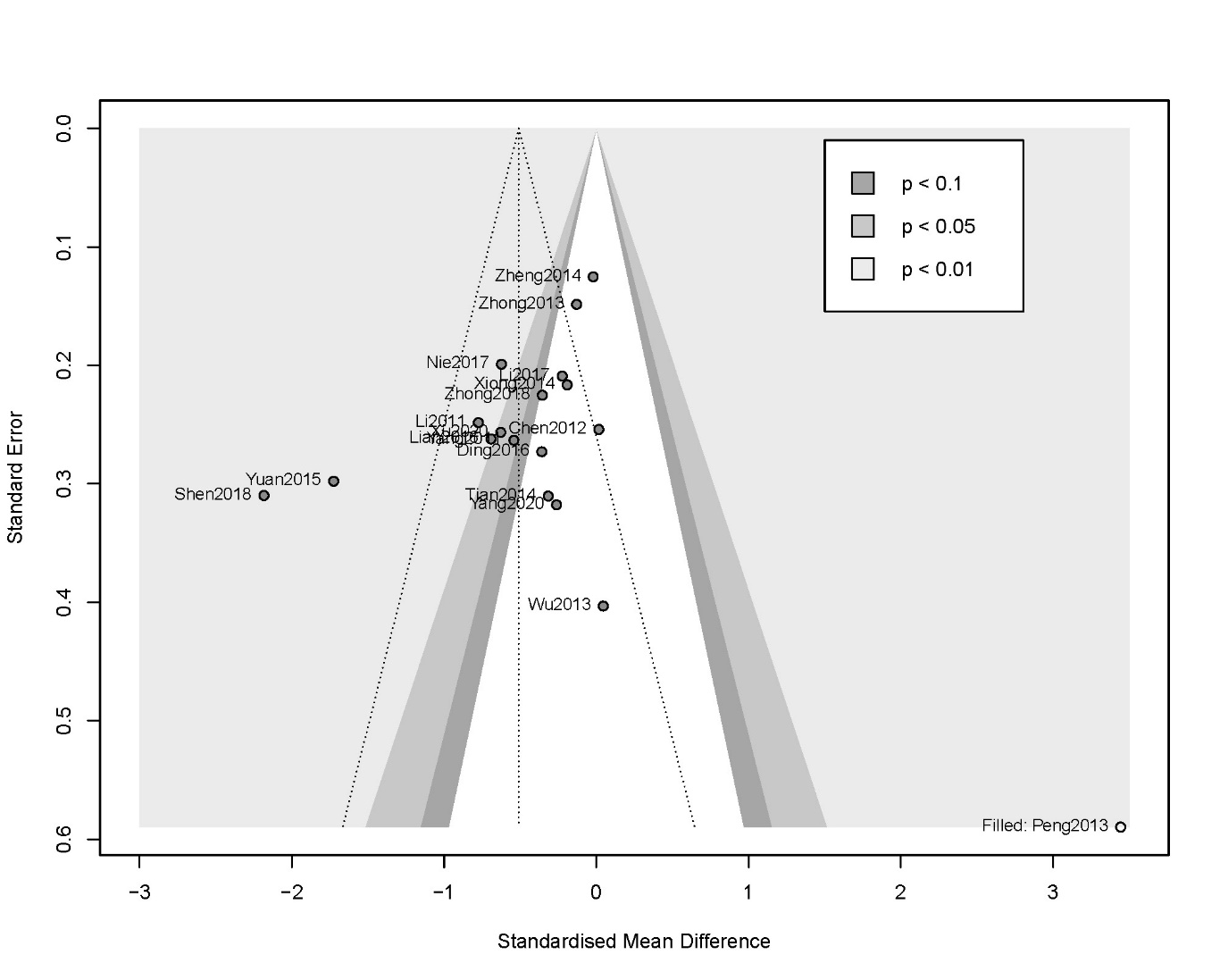
**

**Figure S22 Contour-enhanced funnel plot of depression (Acupuncture VS Pharmacotherapy) after trim-and-fill analysis**

**
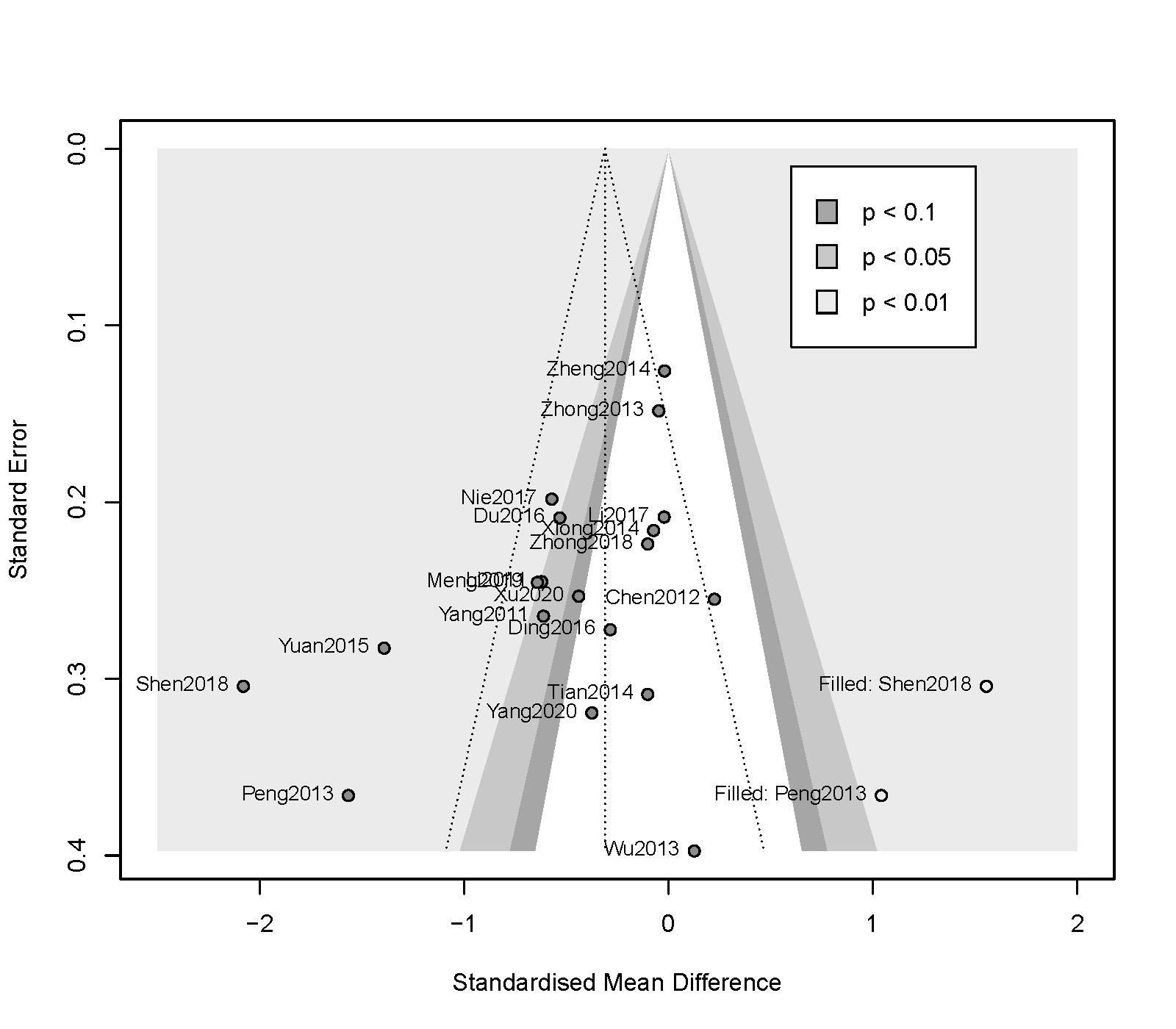
**

| Table S1 PubMed search strategy | |
| --- | --- |
| NO. | Searchs |
| #1 | IBS [Title/Abstract] |
| #2 | Irritable Bowel Syndrome [Title/Abstract] |
| #3 | Irritable Bowel Syndrome [MeSH] |
| #4 | Functional dyspepsia [Title/Abstract] |
| #5 | Functional dyspepsia [MeSH] |
| #6 | functional diarrhoea [Title/Abstract] |
| #7 | functional diarrhoea [MeSH] |
| #8 | functional constipation [Title/Abstract] |
| #9 | functional constipation [MeSH] |
| #10 | #1 OR #2 OR #3 OR #4 OR #5 OR #6 OR #7 OR #8 OR #9 |
| #11 | Acupuncture [MeSH] |
| #12 | Acupuncture [Title/Abstract] |
| #13 | Electroacupuncture [Title/Abstract] |
| #14 | electro-acupuncture [Title/Abstract] |
| #15 | acupuncture therapy [Title/Abstract] |
| #16 | point injection [Title/Abstract] |
| #17 | Acupoint [Title/Abstract] |
| #18 | #11 OR #12 OR #13 OR #14 OR #15 OR #16 OR #17 |
| #19 | Randomized controlled trial [Publication Type] |
| #20 | Randomized [Title/Abstract] |
| #21 | #19 OR #20 |
| #22 | #10 AND # 18 AND #21 |

E: Experimental Group; C: Control Group; MA: Manual acupuncture; EA: Electroacupuncture; SA: Sham acupuncture; NR: Not report; SAS: Self-Rating Anxiety Scale; SDS: Self-Rating depression Scale; HAMA: Hamilton Rating Scale for Anxiety; HAMD: Hamilton Rating Scale for Depression; PHQ-9: Patient Health Questionnaire-9 items; GAD-7: Generalized Anxiety Disorder-7 items

**Table S2 Excluded studies**

| Exclude studies | Reason for exclusion |
| --- | --- |
| Arvidsdotter T, Marklund B, Taft C. Effects of an integrative treatment, therapeutic acupuncture and conventional treatment in alleviating psychological distress in primary care patients--a pragmatic randomized controlled trial. BMC Complement Altern Med. 2013 Nov 7;13:308. doi: 10.1186/1472-6882-13-308. | not meet the inclusion criteria of the disease |
| Cao H M, Deng M J, Liang J B, et al. Thread-embedding Therapy in the Treatment of Irritable Bowel Syndrome of Liver Stagnation and Spleen Deficiency[J]. Shaanxi Journal of Traditional Chinese Medicine, 2018,39(2):262-264. | not meet the inclusion criteria of the acupuncture group |
| Central mechanism of acupuncture treating for functional constipation ChiCTR1800016658 | no specific data reported |
| Chen L, Sun J H, Han G H, et al. Clinical Study on Treatment of Functional Constipation by Deep Needling Tianshu and Abdominal Jie Points Combined with Electroacupuncture[J]. Shanghai Journal of Acupuncture and Moxibustion, 2016,35(03):287-290. | not meet the inclusion criteria of the outcomes |
| Chen Q, Zhou Y, Zhang M, et al. Efficacy Observation of Acupuncture Combined with Salt-partitioned Moxibustion for IBS-D of Spleen Deficiency Pattern[J]. Shanghai Journal of Acupuncture and Moxibustion, 2021,40(4):400-405. | not meet the inclusion criteria of the acupuncture group |
| Chen W C, Ye Y S, Hou X Y, et al. Effect of transcutaneous vagus nerve stimulation on local consistency of brain in patients with functional dyspepsia[J]. Chinese Imaging Journal of Integrated Traditional and Western Medicine, 2021,19(01):6-10. | not meet the inclusion criteria of the acupuncture group |
| Chen X L, Chen X, She L J, et al. Mechanism of transcutaneous electrical acupoint stimulation in improving the symptoms and gastric accommodation in patients with functional dyspepsia[J]. Chinese Journal of Digestion, 2021,41:16-22. | not meet the inclusion criteria of the acupuncture group |
| Chen Y H, Chen X K, Yin X J. Comparison of the therapeutic effects of electroacupuncture and probiotics combined with deanxit in treating diarrhea-predominant irritable bowel syndrome[J]. 2012,32(5):594-598. | data come from the same study included in qualitative synthesis |
| Effect of Acupuncture on Patients With Functional Dyspepsia: a Multi-center, Randomized, Waitlist-controlled Trial. ClinicalTrials.gov Identifier: NCT01921504 | no specific data reported |
| Electrical Stimulation Over Acupuncture Points in Reduction of Rectal Discomfort Distension. ClinicalTrials.gov Identifier: NCT01551654 | no specific data reported |
| Forbes A, Jackson S, Walter C, et al. Acupuncture for irritable bowel syndrome: A blinded placebo-controlled trial[J]. World Journal of Gastroenterology, 2005,11(26):4040-4044. | no specific data reported |
| Geng H, Yang Q H. Observation on Curative Effect of Tongyuan Warming Acupuncture Method, Tongyuan Acupuncture Method and Acupuncture Method on Diarrhea-Type Irritable Bowel Syndrome (Stagnation of Liver and Spleen Type)[J]. Journal of Sichuan of Traditional Chinese Medicine, 2018,36(2):185-188. | not meet the inclusion criteria of the control group |
| Han G H. A Randomized Controlled Study of Deep Needling Tianshu and Abdominal Jie Points for Treatment of Severe Functional Constipation[D]. Nanjing University Of Chinese Medicine, 2014. | not meet the inclusion criteria of the outcomes |
| Huang T. Clinical study on acupuncture treatment of diarrhea-type irritable bowel syndrome[D]. Nanjing University Of Chinese Medicine, 2017. | not meet the inclusion criteria of the outcomes |
| Jiang W. Clinical study of catgut embedding on acupoints for the treatment of liver-stomach discordant functional dyspepsia and postprandial discomfort syndrome[D]. Hubei University of Chinese Medicine, 2020. | not meet the inclusion criteria of the acupuncture group |
| Jin W J. Evaluation of Therapeutic Effect of Transcutaneous Electrical Acupoint Stimulation on Patients with Irritable Bowel Syndrome[J]. Shaanxi Journal of Traditional Chinese Medicine, 2016,37:478-479. | not meet the inclusion criteria of the acupuncture group |
| Kim S K, Lee H, Kuo B, et al. Does realistic clinical acupuncture treatment have an effect on functional dyspepsia?[J]. Gastroenterology, 2015,148(4):S820-S821. | not meet the inclusion criteria of the control group |
| Ko S J, Kim S K, Kim M J, et al. The effect of acupuncture treatment on functional dyspepsia: Pilot study[J]. Integrative Medicine Research, 2015,4(1):20. | not meet the inclusion criteria of the control group |
| Ko S J, Kuo B, Kim S K, et al. Individualized acupuncture for symptom relief in functional dyspepsia: A randomized controlled trial[J]. Journal of Alternative and Complementary Medicine, 2016,22(12):997-1006. | not meet the inclusion criteria of the control group |
| Li L, Li Y, Bao X Y, et al. RCTs of Feiteng Bafa Acupuncture Treating IBS-D of Deficiency of Spleen and Stomach[J]. Journal of Clinical Acupuncture and Moxibustion, 2018,34(6):12-15. | not meet the inclusion criteria of the control group |
| Li R G, Wang W, Jing Q, et al. Effect of Electroacupuncture on Colonic Transfusion and Mental State of Patients with Anxiety and Depression with Irritable Bowel Syndrome: 2016 The 8th National Academic Conference of the Mental Disease Branch of the Chinese Society of Chinese Medicine, Harbin, 2016[C]. | unable to get full-text information |
| Li S L, Zhou C, Sun Y Z. Study on the Application of the Method of Regulating the Mind in the Treatment of Diarrhea-type Irritable Bowel Syndrome[J]. Journal of Clinical Acupuncture and Moxibustion, 2020,36(01):17-20. | not meet the inclusion criteria of the acupuncture group |
| Li W. Intervention effect of electroacupuncture on Shumu points of Dachangchang on patients with functional diarrhea[D]. Hunan University of Chinese Medicine, 2012. | data come from the same study included in qualitative synthesis |
| Li Y B, Zhu Y P, Luo Z Q, et al. Efficiency observation of Anchang Zhixie recipe and acupuncture on irritable bowel syndrome of spleen deficiency and overabundance dampness type[J]. Shanxi Journal of Traditional Chinese Medicine, 2020,36(2):30-32. | not meet the inclusion criteria of the acupuncture group |
| Li Z M. Acupuncture treatment of diarrhea-type irritable bowel syndrome (liver stagnation and spleen deficiency syndrome) clinical research[D]. Liaoning University Of Traditional Chinese Medicine, 2012. | not meet the inclusion criteria of the outcomes |
| Liao W, Wang H, Wang J, et al. Observation on Therapeutic Effect of Acupuncture on Refractory Functional Dyspepsia in Soldiers[J]. People's Military Surgeon, 2012,55(11):1051-1052. | not meet the inclusion criteria of the disease |
| Lin L, Dai R S, Wang Q L, et al. 20 Cases of Chronic Functional Constipation Treated with Acupuncture Combined with Mosapride[J]. Chinese and Foreign Medical Research, 2020,18(36):123-125. | not meet the inclusion criteria of the acupuncture group |
| Liu S S. Observation and Study on Therapeutic Effect of Acupuncture and Moxibustion on Functional Dyspepsia with Mood Disorder[J]. Medical Community, 2020,0(12):132-133. | not meet the inclusion criteria of the outcomes |
| Liu Z Z. Observation on the clinical curative effect of combined acupuncture points in the treatment of functional diarrhea[D]. Changchun University of Chinese Medicine, 2014. | data come from the same study included in qualitative synthesis |
| Luigi G, Vittorio M, Alfredo V, et al. Randomised controlled trial comparing acupuncture with placebo acupuncture for the treatment of irritable bowel syndrome[J]. European Journal of Integrative Medicine, 2012,4:121. | unable to get full-text information |
| Mao W. Observation on the clinical effect of electroacupuncture on constipation-type irritable bowel syndrome[D]. Hubei University of Chinese Medicine, 2018. | not meet the inclusion criteria of the outcomes |
| Mao Y D. Clinical Observation on the Treatment of Functional Dyspepsia with Electroacupuncture and Mosapride[D]. Hubei University of Chinese Medicine, 2014. | not meet the inclusion criteria of the acupuncture group |
| Meng X L, Wang S. Study on the Therapeutic Effect of "Tiao Shen Chang Qing Three Six Nine" Acupuncture Therapy on Functional Constipation[J]. Heilongjiang Journal of Traditional Chinese Medicine, 2019,48(2):213-215. | not meet the inclusion criteria of the control group |
| Peng K M, Luo P. Observation on Therapeutic Effect of Acupuncture and Moxibustion on Functional Dyspepsia Accompanied by Mood Disorder[J]. Journal of Clinical Acupuncture and Moxibustion, 2016,32(06):1-4. | not meet the inclusion criteria of the acupuncture group |
| Peng S F, Shi Z H, Mei Z M, et al. Effect of electroacupuncture at Neiguan and Zusanli on the symptoms, mental state and quality of life of patients with functional dyspepsia: The 21st National Conference on Digestive System Diseases of Integrated Traditional Chinese and Western Medicine, Nanning, 2009[C]. | data come from the same study included in qualitative synthesis |
| Peng S F, Yang J Y, Shi Z H, et al. Electroacupuncture improves gastric motility, autonomic nerve function and mental state in functional dyspepsia[J]. World Chinese Journal of Digestology, 2008,16(36):4105-4109. | no specific data reported |
| Peng S, Yang J, Shi Z. Electroacupuncture improves gastric motility, autonomic nerve activity and psychological state in patients with functional dyspepsia[J]. 2008,16(36):4105-4109. | data come from the same study included in qualitative synthesis |
| Qi R X. Clinical Study on "Promoting Qi and Eliminating Pi" in Treating Post-stroke Dyspepsia (Postprandial Discomfort Syndrome)[D]. Tianjin University of Traditional Chinese Medicine, 2018. | not meet the inclusion criteria of the disease |
| Qiu M X. Study on the Effect and Mechanism of Transcutaneous Electrical Nerve Stimulation at Zusanli Point on Patients with Slow Transit Type Functional Constipation[D]. Huazhong University of Science and Technology, 2009. | not meet the inclusion criteria of the acupuncture group |
| Rafiei R, Ataie M, Ramezani M A, et al. A new acupuncture method for management of irritable bowel syndrome: A randomized double blind clinical trial[J]. Journal of Research in Medical Sciences, 2014,19(10):913-917. | not meet the inclusion criteria of the acupuncture group |
| Reynolds J A, Bland J M, MacPherson H. Acupuncture for irritable bowel syndrome[J]. ACUPUNCTURE IN MEDICINE, 2008,1(26):8-16. | no specific data reported |
| Shang F T. Effects of transcutaneous electroacupuncture therapy on sepsis patients with functional dyspepsia after ICU discharge: a cross-sectional study and randomized controlled trial(ChiCTR1800019475 ). 2018. | not meet the inclusion criteria of the acupuncture group |
| Wang J. Analysis of clinical curative effect of acupuncture on functional bowel disease[D]. Guangzhou University of Chinese Medicine, 2014. | data come from the same study included in qualitative synthesis |
| Wang J. Comparative study of electroacupuncture and prucalopride in the treatment of severe chronic constipation in women[D]. Guangzhou University of Chinese Medicine, 2016. | not meet the inclusion criteria of the outcomes |
| Wang X Y. Clinical Study on Acupuncture Treatment of Functional Dyspepsia with Mood Disorder[J]. Shenzhen Journal of Integrated Traditional Chinese and Western Medicine, 2019,29(12):153-154. | not meet the inclusion criteria of the acupuncture group |
| Wong R K, Fang S Q, Lee T L, et al. Acupuncture therapy improves bloating in functional dyspepsia patients: A randomized controlled trial of sham vs. real acupuncture[J]. Gastroenterology, 2009,136(5):A182. | unable to get full-text information |
| Wu D, Peng T, Rong P J, et al. Efficacy of electro-acupuncture on ear concha in the treatment of diarrhea-type irritable bowel syndrome[J]. World Chinese Medicine, 2021,16(11):1721-1725. | not meet the inclusion criteria of the acupuncture group |
| Wu D, Rong P J, Wang H C, et al. Efficacy Observation on 90 Cases of Functional Dyspepsia Treated by Electroacupuncture at Concha Auriculae[J]. World Chinese Medicine, 2020,15:627-631 | not meet the inclusion criteria of the acupuncture group |
| Wu D, Wang Y, Zhang J L, et al. Transcutaneous auricular vagus nerve stimulation for functional dyspepsia: A randomized controlled trial[J]. World Journal of Acupuncture - Moxibustion, 2021. | data come from the same study included in qualitative synthesis |
| Wu D. Study on the effect and mechanism of ear concha electroacupuncture on functional dyspepsia based on p38MAPK/NF-κB signal pathway[D]. China Academy of Chinese Medical Sciences, 2020. | data come from the same study included in qualitative synthesis |
| Wu J N. A Randomized Controlled Trial of Comparison of the Efficacy of Electroacupuncture at Different Acupoints in the Treatment of Functional Constipation[D]. China Academy of Chinese Medical Sciences, 2013. | data come from the same study included in qualitative synthesis |
| Wu X. Observation on the clinical curative effect of different intensity electroacupuncture in the treatment of functional constipation and preliminary study on its effect on patients' serum metabolic components[D]. Huazhong University of Science and Technology, 2017. | no specific data reported |
| Yang F. Discussion on Clinical Application Value of Tongyuan Warming Acupuncture Method and Acupuncture Method in Diarrhea-type Irritable Bowel Syndrome[J]. Journal of Sichuan of Traditional Chinese Medicine, 2017,35(6):187-190. | not meet the inclusion criteria of the control group |
| Yang J W, Wang L Q, Zou X, et al. Effect of Acupuncture for Postprandial Distress Syndrome: A Randomized Clinical Trial[J]. Annals of internal medicine, 2020,172(12):777-785. | not meet the inclusion criteria of the outcomes |
| Yang J Y, Peng S F, Shi Z H, et al. The influence of electroacupuncture acupoint stimulation on the symptoms, mental state and quality of life of patients with functional dyspepsia: The First International Academic Conference on Digestive Diseases of the Digestive Disease Professional Committee of the World Federation of Chinese Medicine Societies, Beijing , 2010[C]. | data come from the same study included in qualitative synthesis |
| Yang L J, Wang X X, Li B, et al. Effect of Acupuncture on the Acupoint Pain Threshold in Patients with Irritable Bowel Syndrome[J]. Shanghai Journal of Acupuncture and Moxibustion, 2018,37:1030-1036. | not meet the inclusion criteria of the outcomes |
| Yang T. Clinical Observation on Acupuncture of "Shenque Bazhen" in Treating Irritable Bowel Syndrome of Spleen Deficiency and Dampness[D]. Chengdu University of TCM, 2020. | not meet the inclusion criteria of the acupuncture group |
| Ye K S, Chen M X, Jin M, et al. Application of TCM Syndrome Scale to Evaluate the Efficacy of TEAS in the Treatment of Functional Dyspepsia[J]. Chinese Journal of Traditional Medical Science and Technology, 2013,20:507-508. | not meet the inclusion criteria of the acupuncture group |
| Yin P, Gao N, Dong B, et al. Efficacy and safety of acupuncture on the treatment of functional constipation: study protocol for a randomized controlled trial[J]. 2019,29(2):145-152. | protocol |
| Yu C D. Analysis of TCM Syndrome Types of Refractory Functional Dyspepsia and Observation of Acupuncture Efficacy[D]. Hubei University of Chinese Medicine, 2020. | not meet the inclusion criteria of the outcomes |
| Yu L M, Zhang Y L, Wang Y W, et al. Umbilicus acupuncture for treatment of diarrhea-type irritable bowel syndrome: Efficacy and impact on brain-gut peptides[J]. World Chinese Journal of Digestology, 2020,28(13):538-543. | not meet the inclusion criteria of the acupuncture group |
| Zhan D W. Effect of acupuncture with the method of soothing the liver and strengthening the spleen on the changes of serum 5-HT, NPY and CGRP levels in patients with diarrheal irritable bowel syndrome[D]. Nanjing University Of Chinese Medicine, 2013. | not meet the inclusion criteria of the outcomes |
| Zhang B Y. Preliminary comparison of the curative effect of different acupoints in relieving symptoms of functional diarrhea[D]. Beijing University of Chinese Medicine, 2014. | data come from the same study included in qualitative synthesis |
| Zhang L W. A Randomized Controlled Preliminary Clinical Study on Acupuncture "Old Ten Needles" Modified and Subtracted Recipe in Treating Postprandial Discomfort Syndrome[D]. Shandong university of traditional chinese medicine , 2017. | not meet the inclusion criteria of the outcomes |
| Zhang W, Chen Y. Effect of Acupuncture on Psychological Factors and Serum Ghrelin Level in Patients with Functional Dyspepsia[J]. Shanghai Journal of Acupuncture and Moxibustion, 2018,37(01):37-41. | not meet the inclusion criteria of the outcomes |
| Zhang X, Ding M, Feng Y, et al. Acupuncture with Du's heat-reinforcing method for diarrhea-predominant irritable bowel syndrome:a randomized controlled trial[J]. Journal of Acupuncture and Tuina Science, 2019,17:124-130. | not meet the inclusion criteria of the outcomes |
| Zhou J. Observation on the effect of electroacupuncture on severe chronic functional constipation in women[D]. Beijing University of Chinese Medicine, 2016. | not meet the inclusion criteria of the outcomes |
| Zhou S F. Observation of the effect of percutaneous vagus nerve stimulation on functional dyspepsia and fMRI study of central mechanism[D]. Guangzhou University of Chinese Medicine, 2020. | not meet the inclusion criteria of the acupuncture group |
| Zhou Y. a clinical study on acupuncture and moxibustion treatment of slow transit constipation based on brain-gut axis theory[D]. Yunnan University of Chinese Medicine, 2019. | not meet the inclusion criteria of the disease |
| Zhu R X. Observation on the clinical curative effect of Dachangjing, Fu-organs and acupoints in the treatment of functional constipation[D]. Hunan University of Chinese Medicine, 2012. | data come from the same study included in qualitative synthesis |
